# Epoxide hydrolysis as a model system for understanding flux through a branched reaction scheme

**DOI:** 10.1107/S2052252518003573

**Published:** 2018-03-22

**Authors:** Åsa Janfalk Carlsson, Paul Bauer, Doreen Dobritzsch, Shina C. L. Kamerlin, Mikael Widersten

**Affiliations:** a Department of Chemistry, BMC, Uppsala University, Box 576, 751 23 Uppsala, Sweden; b Science for Life Laboratory, Department of Cell and Molecular Biology, Uppsala University, BMC Box 596, 751 24 Uppsala, Sweden

**Keywords:** epoxide hydrolase, stereoselectivity, empirical valence-bond simulations, biocatalysis, reaction flux, *trans*-methylstyrene oxide

## Abstract

The epoxide hydrolase StEH1 catalyzes the hydrolysis of *trans*-methylstyrene oxide to 1-phenyl­propane-1,2-diol. The (*S*,*S*)-epoxide is exclusively transformed into the (1*R*,2*S*)-diol, while hydrolysis of the (*R*,*R*)-epoxide results in a mixture of product enantiomers. In order to understand the differences in the stereoconfigurations of the products, the reactions were studied kinetically during both the pre-steady-state and steady-state phases. A number of closely related StEH1 variants were analyzed in parallel, and the results were rationalized by structure–activity analysis using the available crystal structures of all tested enzyme variants. Finally, empirical valence-bond simulations were performed in order to provide additional insight into the observed kinetic behaviour and ratios of the diol product enantiomers. These combined data allow us to present a model for the flux through the catalyzed reactions. With the (*R*,*R*)-epoxide, ring opening may occur at either C atom and with similar energy barriers for hydrolysis, resulting in a mixture of diol enantiomer products. However, with the (*S*,*S*)-epoxide, although either epoxide C atom may react to form the covalent enzyme intermediate, only the *pro*-(*R*,*S*) alkylenzyme is amenable to subsequent hydrolysis. Previously contradictory observations from kinetics experiments as well as product ratios can therefore now be explained for this biocatalytically relevant enzyme.

## Abstract 2

Kinetic rates and computer simulations can explain regioselectivity and stereoconfiguration in the products of enzyme-catalyzed epoxide hydrolysis.

## 1. Introduction   

**Figure 1:**
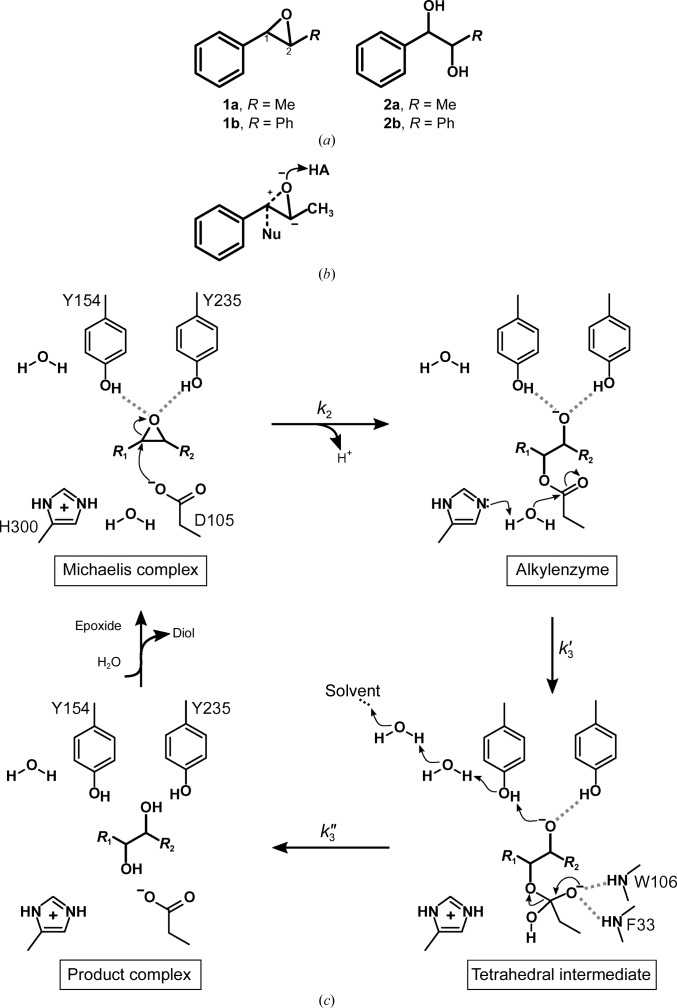
(*a*) **1a**, *trans*-methylstyrene oxide; **1b**, *trans*-stilbene oxide. **2a** and **2b** are the respective hydrolysis products. (*b*) Epoxide ring opening following nucleophilic attack. C—O bond breakage is facilitated by acid stabilization of the leaving-group oxyanion and electron donation to the electrophilic C atom. (*c*) Mechanism of StEH1-catalyzed epoxide hydrolysis. The carboxylate side chain of Asp105 performs nucleophilic attack on one of the oxirane C atoms. The formed oxyanion is stabilized by the phenol groups of Tyr154 and Tyr235. This alkylenzyme intermediate is subsequently hydrolyzed by a base-activated (His300) water. See Fig. 4 ▸ for the location of these catalytic residues within the active site. The transient presence of the alkylenzyme can be detected from quenching of the intrinsic protein fluorescence, allowing estimation of its rates of formation (*k*_2_) and decay (*k*_3_). The rate of formation of the tetrahedral intermediate (*k*′_3_ in the figure) is expected to be rate-determining for hydrolysis of the alkyl­enzyme, due to the intrinsic instability of this intermediate (*k*′′_3_ ≫ *k*′_3_). Note that, formally, *k*_3_ measures all steps involved from hydrolysis of the alkyl­enzyme to product release; hence, this has been divided into *k*′_3_ and *k*′′_3_ for clarity.

Epoxides are important components in the toolbox of chiral building blocks for asymmetric synthesis (Santaniello *et al.*, 1992 ▸). Therefore, it would be of great value to be able to control epoxide ring opening, and thus also to predict the stereoconfigurations of the products formed. In aqueous solution, the regioselectivity of ring opening by a nucleophile depends on the electron affinity of the substituents and on steric effects. For instance, the hydrolysis of *trans*-methylstyrene oxide (**1a**; Fig. 1 ▸ *a*) is expected to occur *via* breakage of the bond between the benzylic C atom (C1) and the epoxide O atom. This prediction is based on electron donation from the phenyl substituent, which stabilizes the buildup of positive charge on the attacked C atom (Fig. 1 ▸ *b*; Parker & Isaacs, 1959 ▸). The reaction is further facilitated by protonation of the leaving-group oxide. However, making such regioselectivity predictions is far less trivial in an enzyme-catalyzed epoxide ring-opening reaction, since the chiral nature of the active site and its particular microenvironment steer the reaction in directions that are uncoupled from the intrinsic chemical reactivities (Monterde *et al.*, 2004 ▸) of the epoxide substrates. To correctly predict the resulting stereoselectivity therefore becomes a major and often unmet challenge in itself.

Epoxide hydrolase 1 from potato (StEH1; Stapleton *et al.*, 1994 ▸) catalyzes the hydrolytic ring-opening reactions of monosubstituted and *trans*-disubstituted alkyl and aryl epoxides by an S_N_2 mechanism, resulting in inversion of the stereoconfiguration when the attacked epoxide C atom is asymmetric. The catalytic mechanism is that of a generic α/β-hydrolase (Heikinheimo *et al.*, 1999 ▸), involving a combination of nucleophilic and acid/base catalysis: the carboxylate of Asp105 attacks one of the electrophilic epoxide C atoms, forming a negatively charged covalent alkylenzyme intermediate which is stabilized by two tyrosine phenols (Fig. 1 ▸ *c*). This ester intermediate is subsequently hydrolyzed by a general-base-activated water molecule, where the side chain of the active-site histidine, His300, acts as a general base (Pinot *et al.*, 1995 ▸; Tzeng *et al.*, 1996 ▸, 1998 ▸; Elfström & Widersten, 2005 ▸; Widersten *et al.*, 2010 ▸).

The StEH1-catalyzed hydrolysis of (*S*,*S*)-**1a** proceeds with high regioselectivity for the benzylic C atom, which is in accordance with what would be expected from the intrinsic reactivities (Table 1 ▸; Lindberg *et al.*, 2008 ▸; Lindberg, Ahmad, 2010 ▸). The regioselectivity in the hydrolysis of (*R*,*R*)-**1a**, however, is less strict, although with a modest preference for ring opening at the benzylic epoxide C atom. The hydrolysis of the epoxide thus generates both the (1*R*,2*S*)-**2a** and (1*S*,2*R*)-**2a** diol enantiomers, and the corresponding regiopreference is sensitive to environmental conditions such as pH and temperature (Lindberg, de la Fuente Revenga *et al.*, 2010 ▸). This enantiomer-dependent regio­preference is not due to trivial causes such as steric clashes in the active site (Lindberg, de la Fuente Revenga *et al.*, 2010 ▸; Reetz *et al.*, 2009 ▸). However, since enantioconvergence is a highly desired feature in asymmetric synthesis, an increased understanding of the underlying mechanisms is both motivated and necessary. It is known, for example, that StEH1 catalyzes the hydrolysis of styrene oxide (*R* = H in Fig. 1 ▸ *a*), resulting in enantioconvergence to (*R*)-phenylethanediol (Monterde *et al.*, 2004 ▸; Janfalk Carlsson *et al.*, 2012 ▸). This makes StEH1 an excellent model system for the design of novel enantioconvergent biocatalysts.

**Figure 2:**
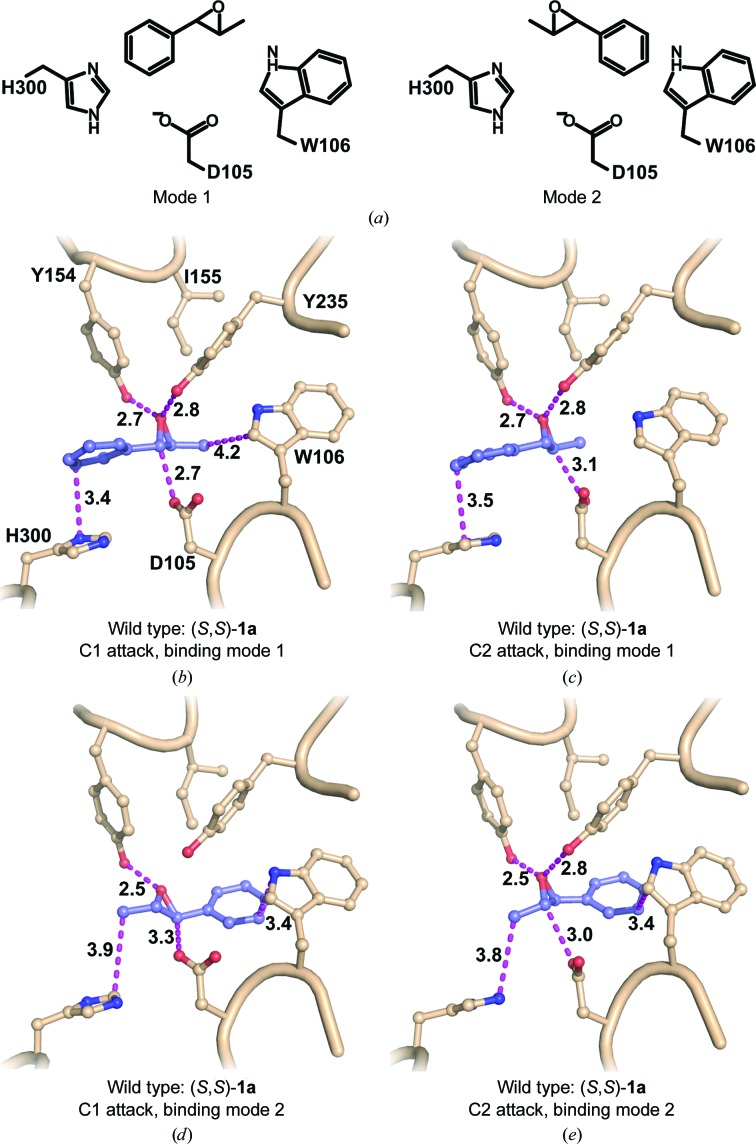
(*a*) Schematic illustration of the two tested modes of binding **1a** to the active site of StEH1. In mode 1, the phenyl ring of the substrate interacts with His300, while in mode 2 the interaction instead involves Trp106 (or Leu106 in the R-C1B1 variant). (*b*–*e*) Representative Michaelis complexes between wild-type StEH1 and (*S*,*S*)-**1a** bound in mode 1 (*b*, *c*) or mode 2 (*d*, *e*) with epoxide ring opening at C1 (*b*, *d*) or C2 (*c*, *e*). The structures correspond to the clusters of the Michaelis complex as obtained by EVB simulations of the epoxide ring-opening step and were constructed with *PyMOL* v.1.8.7 (Schrödinger).

In a recent computational study using the empirical valence-bond (EVB) approach, we managed to both qualitatively and quantitatively mimic the experimentally determined enantiomeric ratios of the hydrolysis products (Bauer *et al.*, 2016 ▸). An important insight provided by this study was that despite the completely different regioselectivities of epoxide ring opening for the different enantiomers, both enantiomers displayed the same preferred substrate-binding mode (of two possible binding modes; see Fig. 2 ▸). That is, rather than being driven by radical changes in binding mode, the differences in selectivity appeared to be owing to subtle variations in binding interactions between the enzyme and the substrate for the different enantiomers. In combination with differences in the interactions with active-site water molecules, this resulted in distinct configurations for nucleophile attack, ultimately leading to enantioconvergence to the (*R*)-diol product.

**Figure 3:**
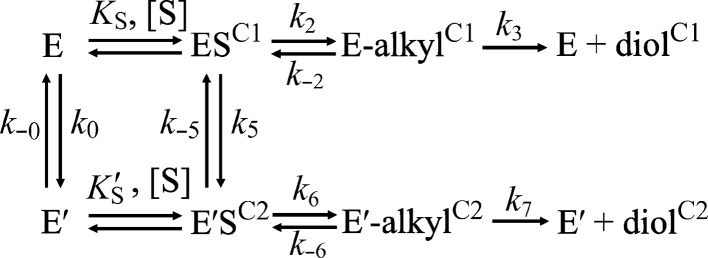
Model of the kinetic mechanism for epoxide hydrolysis catalyzed by StEH1. The model is based on data from pre-steady-state and steady-state kinetics and the enantiomeric ratios of the product diols.

The kinetic scheme for the hydrolysis of styrene oxide can be described by a simple linear Michaelis–Menten model, and the formation and decay of only one alkylenzyme can be observed during the pre-steady-state phase of the reaction (Bauer *et al.*, 2016 ▸). However, the case of the hydrolysis of **1a** is more complex, and the fact that two enantiomeric product diols can be formed from an optically pure starting epoxide by necessity invokes a branched reaction scheme. We have proposed a model (Fig. 3 ▸) that builds on earlier experimental results (Elfström & Widersten, 2005 ▸, 2006 ▸; Thomaeus *et al.*, 2007 ▸; Lindberg *et al.*, 2008 ▸; Lindberg, Ahmad *et al.*, 2010 ▸; Lindberg, de la Fuente Revenga *et al.*, 2010 ▸; Widersten *et al.*, 2010 ▸). The model invokes a substrate-independent conformational step (E to E′) as well as reversible isomerizations of the respective Michaelis complexes (ES to E′S). The catalytic mechanism includes covalent alkylenzyme(s) (E-alkyl and E′-alkyl, respectively), which are considered not to be interconvertible. If the formed alkylenzyme is readily hydrolyzed to a diol, the regioselectivity of the nucleophilic attack on the epoxide ring also decides the product configuration. However, if the barrier for hydrolysis is higher than that for decomposition to the Michaelis complex(es), then interconversion between ES and E′S is conceivable. Hence, an alkylenzyme with the opposite configuration may be formed, albeit at a lower steady-state concentration. If, however, this (less favoured) alkyl­enzyme intermediate is efficiently hydrolyzed, the diol product will inherit its configuration. This model of the different states of productive enzyme–substrate complexes describes the simplest cases and does not explicitly include the concept of different substrate-binding modes within the active sites of either E or E′. The assumption is that the different binding modes are rapidly equilibrated on a timescale that is not rate-limiting for catalysis.

Experimental validation of the model can be achieved by monitoring the transient buildup and decay of the respective alkylenzymes and by measuring the enantiomer ratios of the product diols. If one alternative pathway leading to product dominates, or if the buildup of one of the alkylenzymes is low in comparison to the other, the model reduces to a simple linear Michaelis–Menten mechanism. It follows, therefore, that even if the kinetics *appear* to describe a linear mechanism, this does not *prove* the formation of a single product enantiomer. The diol products of either enantiomer of *trans*-stilbene oxide (**1b**) will in all cases be *meso*-hydrobenzoin (**2b**), and therefore product analysis neither supports nor disproves the proposed model.

Using the epoxide **1a** as a substrate, however, allows the kinetic model to also be tested experimentally, since the different product enantiomers are readily individually quantifiable. As mentioned above, the StEH1-catalyzed hydrolysis of (*S*,*S*)-**1a** produces only (1*R*,2*S*)-**2a**, even though two transient rates with distinct amplitudes are detected during the pre-steady state phase of the reaction, suggesting the formation of two distinct alkylenzyme intermediates. It was originally speculated that only one of the alkylenzymes was hydrolyzed to form product (for unknown reasons; Lindberg *et al.*, 2008 ▸). The second rate of alkylation could also come from a different substrate-binding mode, allowing a different rate but with the same regioselectivity. In contrast, the pre-steady-state phase of the hydrolysis of the (*R*,*R*)-enantiomer of **1a** displays only one amplitude, indicating that a single alkylenzyme intermediate is being formed, although a mixture of diol enantiomers are produced (see the data in Table 1 ▸).

**Figure 4:**
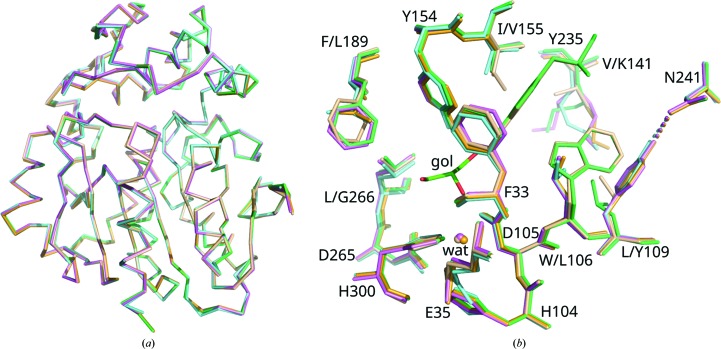
Structures of the enzyme variants analyzed in this work. (*a*) Superposition of the C^α^ traces of wild-type StEH1 (wheat) and the variants R-C1 (lime green), R-C1B1 (violet), R-C1B1D33 (aquamarine) and R-C1B1D33E6 (orange). (*b*) Overlay of active-site residues, including those that differ between the different enzyme variants. The catalytic residues are Asp105 (nucleophile), His300 (general base), Asp265 (charge relay) and Tyr154 and Tyr235 (acids) (see Fig. 1 ▸ for the full reaction mechanism). Glu35 and His104 are auxiliary catalytic residues. The hydrolytic water (wat) is also shown. A glycerol molecule (gol) bound in the active site of R-C1 has been included to show the substrate-binding pocket. The residue replacements are given in Table 2 ▸. The modelled side-chain conformations of Lys141 should be considered arbitrary owing to a lack of clear electron density beyond C^γ^. This figure was constructed from the atomic coordinates in PDB entries 2cjp (wild type; Mowbray *et al.*, 2006 ▸), 4uhb (R-C1; Janfalk Carlsson *et al.*, 2016 ▸), 4ufn (R-C1B1; Bauer *et al.*, 2016 ▸), 4ufp (R-C1B1D33; Janfalk Carlsson *et al.*, 2016 ▸) and 4ufo (R-­C1B1D33E6; Janfalk Carlsson *et al.*, 2016 ▸) using *PyMOL* v.1.8.7.

In the present work, our goal is to provide a general explanation for these observations and for the stereoselectivities exhibited by this enzyme. To address this goal, we have both experimentally and computationally analyzed wild-type StEH1 together with four related laboratory-evolved StEH1 offspring. These enzyme variants were originally isolated for their ability to catalyze the production of enantio-enriched (2*R*)-3-phenylpropane-1,2-diol from a racemic mixture of benzyloxirane (Gurell & Widersten, 2010 ▸; Janfalk Carlsson *et al.*, 2012 ▸, 2016 ▸). We have utilized them, together with the wild-type enzyme, as a series of structurally closely related isoenzymes (Fig. 4 ▸). Together, they represent excellent models to probe the linkage between changes in active-site structure and the corresponding effects of these changes on the observed substrate selectivity.

Epoxides **1a** and **1b** were specifically chosen as model substrates since they also allow data to be gathered during the transient pre-steady-state phase of the reactions. The experimentally determined rates could thus complement theoretical calculations and protein structure analyses. StEH1 is also unusually well suited as a model enzyme system for clarifying some fundamental aspects of enzyme catalysis and substrate recognition because (i) the chemical mechanism is well understood and its generality increases the scope of possible applicable related systems, as highly similar catalytic mechanisms are found in a wide range of hydrolytic enzymes, representing both related (α/β-hydrolases) as well as more structurally unrelated systems (for example enzymes such as the pancreatic proteases and subtilisin); (ii) the different alternative mechanistic possibilities for epoxide ring opening provide opportunities for explaining enantioselectivity and regioselectivity in the transformation of asymmetric substrates and, finally; (iii) crystal structures have been solved for all of the isolated variants (Mowbray *et al.*, 2006 ▸; Bauer *et al.*, 2016 ▸; Janfalk Carlsson *et al.*, 2016 ▸). To further rationalize the functional data, we have performed detailed EVB calculations on the hydrolysis reaction catalyzed by both the wild-type enzyme and the variants studied here. Together, our results allow us to construct a model for flux through a branched reaction scheme that is able to explain both previous contradictory observations from the kinetic experiments and the unusual product ratios that are observed.

## 2. Materials and experimental details   

### 2.1. Enzyme expression and purification   

Wild-type StEH1 and variants were expressed in *Escherichia coli* XL1-Blue (Stratagene) cells carrying a plasmid with genes coding for the GroEL/ES chaperonins (Dale *et al.*, 1994 ▸) and were purified by Ni^2+^–IMAC and size-exclusion chromatography as described previously (Elfström & Widersten, 2005 ▸). Protein concentrations were determined from the UV absorption at 280 nm using an extinction coefficient based on the value for wild-type StEH1 (Elfström & Widersten, 2005 ▸). The extinction coefficients for the R-C1B1, R-C1B1D33 and R-C1B1D33E6 variants were adjusted as described in (1), 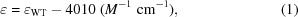
to correct for the W106L and L109Y substitutions in these proteins.

### 2.2. Enzyme kinetics   

The current working model of the kinetic mechanism for this enzyme is shown in Fig. 3 ▸ and is based on previous analysis of kinetics and product ratios. Rate constants are numbered accordingly.

#### 2.2.1. Steady-state kinetics   

Initial rates of hydrolysis of **1b** (Fig. 1 ▸ *a*) were recorded by following the decrease in absorbance at 229 nm (Wixtrom & Hammock, 1988 ▸), reflecting epoxide depletion. Reaction conditions were as described previously (Elfström & Widersten, 2005 ▸), with assays being performed at pH 6.8 and 30°C. Steady-state parameters were determined by nonlinear regression of the Michaelis–Menten equation to the experimental data using *SIMFIT* (http://www.simfit.org.uk).

#### 2.2.2. Pre-steady-state kinetics: multiple-turnover conditions   

**Figure 5:**
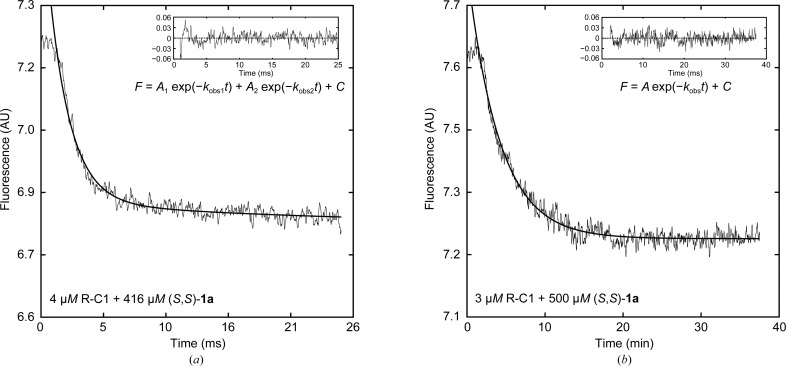

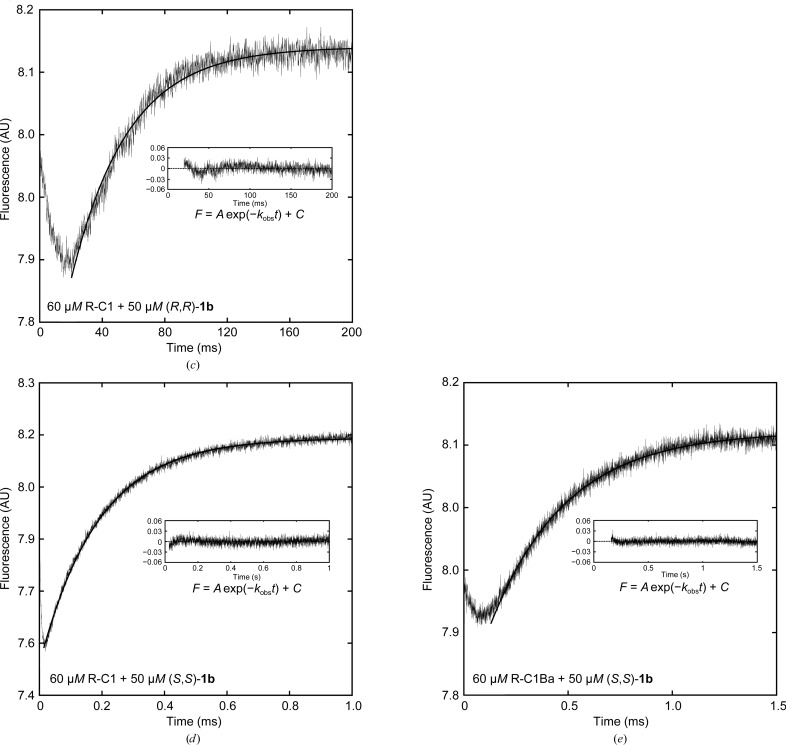
Example averaged fluorescence traces recorded under pseudo-first-order, multiple-turnover conditions (*a*, *b*) and under single-turnover conditions at near-saturating concentrations of **1b** (*c*, *d*, *e*). (*a*) Quenching of the intrinsic protein fluorescence during the pre-steady-state phase of R-C1-catalyzed hydrolysis of (*S*,*S*)-**1a**. The averaged traces were fitted to (3), yielding two observed rates (*k*_obs_). The concentrations of substrate and enzyme are shown on the graph. (*b*) Transient fluorescence decay during R-C1B1-catalyzed hydrolysis of (*S*,*S*)-**1a**. A first-order exponential was fitted to the data (2). (*c*, *d*, *e*) Recovery of protein fluorescence under single-turnover conditions after R-C1-catalyzed hydrolysis of (*R*,*R*)-**1b** (*c*) and (*S*,*S*)-**1b** (*d*) and R-C1B1-catalyzed hydrolysis of (*S*,*S*)-**1b** (*e*), respectively. Solid lines are fits of (2) to the experimental data. The insets show the residuals of the fitted models.

The buildup of steady-state levels of alkylenzyme intermediates formed during the catalyzed hydrolysis of either enantiomer of **1a** and **1b** was followed by monitoring the decrease in the intrinsic tryptophan (wild type and R-C1) or tyrosine (R-C1B1, R-C1B1D33 and R-C1B1D33E6) fluorescence of the enzyme, as described previously (Elfström & Widersten, 2005 ▸). To detect tyrosine fluorescence, an excitation wavelength of 274 nm was applied and light emitted above 305 nm was detected. The apparent rates, *k*_obs_, were determined by fitting either a single exponential function with a floating endpoint,
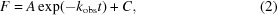
or a double exponential function with a floating endpoint,
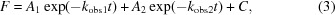
to the averaged progression curves. Averages of at least six traces were used in all cases. When used, the validity of the higher-order equation was verified by an *F*-test. The substrate concentrations were 30–1500 and 3–100 µ*M* enantiopure **1a** and **1b**, respectively. The enzyme concentrations were kept more than tenfold lower than the substrate concentration at all times in order to ensure pseudo-first-order reaction conditions. Example averaged traces of fluorescence quenching are shown in Figs. 5 ▸(*a*) and 5 ▸(*b*). Parameter values were obtained after fitting the determined *k*_obs_ values to either (4) or (5), where the latter describes a two-step process composed of a substrate-independent transition followed by a considerably faster step (Fersht, 1999 ▸). In the current model, the substrate-independent step is the E to E′ transition, with rate sums of *k*_0_ + *k*_−0_, followed by rapid equilibration of the respective Michaelis complexes as described in Fig. 3 ▸.
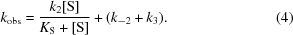


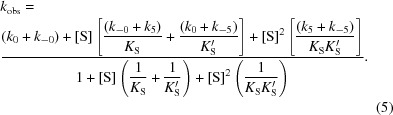


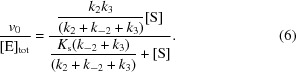



**Figure 6:**
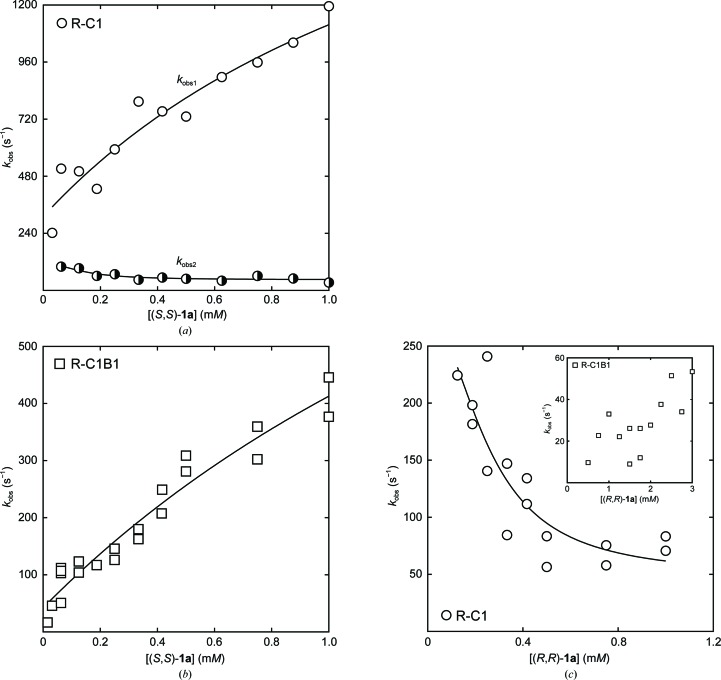
Example curve-fitting of the observed transient rates of alkylenzyme formation and decay. (*a*, *b*) The substrate-dependence of the observed transient rates, *k*_obs_, in the hydrolysis of (*S*,*S*)-**1a** catalyzed by (*a*) R-C1 and (*b*) R-C1B1. In the case of R-C1, the obtained fluorescence traces were fitted to a second-order exponential (3) to yield two observed rates (unfilled and half-filled circles, respectively), while the R-C1B1-catalyzed rates could be fitted to a first-order exponential (2). The substrate-dependencies of the respective *k*_obs_ values were fitted to (4) in the cases of a hyperbolic dependency [unfilled symbols in (*a*) and (*b*)] and to (5) in the case of a decreasing *k*_obs_ with an increasing concentration of substrate [half-filled symbols in (*a*)]. (*c*) The substrate-dependence of the observed transient rates, *k*_obs_, in the hydrolysis of (*R*,*R*)-**1a** catalyzed by R-C1 and R-C1B1 (inset). The observed concentration-dependency in the R-C1B1-catalyzed reaction was inconclusive and no fitting was performed. In the case of R-C1, the obtained *k*_obs_ values were fitted to (5). See §[Sec sec2]2 for details.

In cases where *k*_obs_ displayed a hyperbolic substrate-concentration dependence, *k*_2_ and *K*_S_ were determined after fitting (4) to the observed rates. In cases where the values of *K*_S_ were considerably larger than the solubility limit of the substrate, the estimated values of *k*_2_ and *K*_S_ consequently show large standard errors owing to the required extrapolation (Table 1 ▸). *k*_−2_ and *k*_3_ were calculated from the determined value of their sum (at [S] = 0), applying the derived expression for *k*_cat_ (the numerator in equation 6). The relatively large error margins in the estimated values are owing to the standard errors from the regression analysis connected with the values of *k*_2_ and (*k*_−2_ + *k*_3_). Values for *k*_2_, *k*_−2_ and *k*_3_ were calculated from the experimentally determined value of (*k*_−2_ + *k*_3_) and from the derived expressions for *k*_cat_ and *k*_cat_/*K*_m_ and their determined values. In cases where *k*_obs_ decreased with increasing [S], (5) was fitted to extract the sum of *k*_5_ + *k*_−5_ (Lindberg *et al.*, 2008 ▸; Fig. 6 ▸).

#### 2.2.3. Pre-steady state kinetics: single-turnover conditions   

The rate of hydrolysis, *k*_3_, was directly determined by measuring the tryptophan or tyrosine fluorescence recovery following the alkylation step. This experiment was only performed with the enzyme variants in which a sufficiently low *K*_S_ **^1b^** allowed fulfilment of the criteria [S] and [E] ≫ *K*_S_ without exceeding the solubility limit of the substrate, so that [S]_tot_ ≃ [ES]. Hence, the recorded observed rate of fluorescence recovery reflected the rate of the process ES→E+P and thus *k*_obs_ ≃ *k*_3_. The enzyme concentrations used were in all cases 60 µ*M* in the presence of 50 µ*M* substrate, except for the hydrolysis of (*R*,*R*)-**1b** catalyzed by the wild type, where 115 µ*M* enzyme was mixed with 100 µ*M* substrate. These concentrations correspond to 5 × *K*_S_^(*R*,*R*)-**1b**^ and 9 × *K*_S_^(*S*,*S*)-**1b**^ for the wild-type enzyme, 3.8 × *K*_S_^(*R*,*R*)-**1b**^ and 3.1 × *K*_S_^(*S*,*S*)-**1b**^ for R-C1 and 2.4 × *K*_S_^(*S*,*S*)-**1b**^ for R-C1B1. Example averaged traces are presented in Figs. 5 ▸(*c*), 5 ▸(*d*) and 5 ▸(*e*).

### 2.3. Empirical valence-bond calculations   

All empirical valence-bond (EVB; Warshel & Weiss, 1980 ▸; Warshel, 1980 ▸) calculations in the present study were performed using a similar protocol to that used in our previous work on the StEH1-catalyzed hydrolysis of styrene oxide and *trans*-stilbene oxide (Amrein *et al.*, 2015 ▸; Bauer *et al.*, 2016 ▸). Full details of the simulations, as well as all EVB parameters used in the present work, are provided as Supporting Information. In brief, the EVB approach employs valence-bond (VB) theory to model chemical reactivity using a force-field description of different reacting states, with the propagation of the reaction being achieved using linear mixing of the potentials for the different states that are described. The use of Morse rather than harmonic potentials to describe bonds that are broken or formed during the reaction allows for a physically meaningful description of chemical reactivity, and this classical description is then moved to a quantum-chemical framework through the construction of an *n* × *n* Hamiltonian matrix based on the energies of the different reacting states, where *n* denotes the number of reacting states (for details, see Warshel, 1980 ▸; Shurki *et al.*, 2015 ▸).

As EVB is a parameterized approach, the quality of the results provided by this approach depends on two things: (i) the quality of the parameterization used in the work (and thus the resulting EVB potentials) and (ii) the amount of conformational sampling performed and whether this is sufficient to obtain convergent results. A well parameterized EVB potential with adequate conformational sampling can easily give errors of less than 1 kcal mol^−1^ on the calculated energetics compared with experiment (see, for example, Barrozo *et al.*, 2015 ▸; Blaha-Nelson *et al.*, 2017 ▸; Kulkarni *et al.*, 2017 ▸). In the case of the computational data presented in this work, obtaining reliable absolute energies for each enantiomer through calculations is difficult owing to the uncertainities in the energetics for the corresponding uncatalyzed reaction in aqueous solution, as also discussed in Amrein *et al.* (2015 ▸) and Bauer *et al.* (2016 ▸), hence the larger deviations between the experimental and calculated values. However, the standard deviations on each calculated value (0.7 kcal mol^−1^ or less) are smaller than the calculated differences between the different enantiomers. Therefore, despite the limitations when considering the absolute values from the calculations, the relative values can still be compared in a meaningful way.

The EVB simulations of the hydrolysis of **1a** by wild-type StEH1 and the R-C1 variant were performed using the two different proposed binding modes for (*R*,*R*)-**1a** and (*S*,*S*)-**1a** illustrated in Fig. 2 ▸ and shown using the valence-bond description in Supplementary Fig. S1. Specifically, we have modelled here first the nucleophilic attack of the Asp105 side chain on substrate **1a**, followed by the subsequent activation of a water molecule to form the tetrahedral reaction intermediate that will ultimately decompose to yield the final product diol. As this final step is likely to be extremely fast (McClelland *et al.*, 1990 ▸) we have not modelled it in the present work, focusing our computational resources instead on the steps that are likely to be rate-limiting.

All systems were initially equilibrated for 10 ns using standard molecular dynamics to provide starting points for subsequent EVB simulations. The EVB free-energy perturbation/umbrella sampling (EVB-FEP/US) procedure was then performed in 51 mapping frames of 200 ps in length to give a total of 10.2 ns of simulation time per trajectory. The EVB simulations were performed 30 times for each system, leading to a total simulation time of 14.6 µs over all systems, taking into account both the initial equilibration and subsequent EVB simulations. The EVB simulations of the enzyme-catalyzed reactions were based on the crystal structures of wild-type StEH1 (PDB entry 2cjp; Mowbray *et al.*, 2006 ▸) as well as the R-C1 variant (PDB entry 4uhb; Janfalk Carlsson *et al.*, 2016 ▸). The corresponding uncatalyzed reaction in aqueous solution was modelled by truncating the reacting atoms to propionate, methylimidazole and **1a**, and calibrated to high-level quantum-chemical calculations using density functional theory, as in our previous work on this enzyme (Amrein *et al.*, 2015 ▸; Bauer *et al.*, 2016 ▸). All MD and EVB simulations were performed using the *Q* simulation package (Marelius *et al.*, 1998 ▸) and the OPLS-AA force field (Jorgensen *et al.*, 1996 ▸). For further simulation details, including an in-depth description of the system setup, we refer the reader to the Supporting Information.

All subsequent energy analysis was performed using the *QCalc* module of *Q* (Marelius *et al.*, 1998 ▸), and all structural analysis was performed using *GROMACS* 5.0.5 (Abraham *et al.*, 2015 ▸). All root-mean-square deviation (r.m.s.d.) and clustering calculations were performed without mass weighting, and r.m.s.d. calculations were performed relative to the initial crystal structures in complex with the substrate.

## 3. Results and discussion   

**Figure 7:**
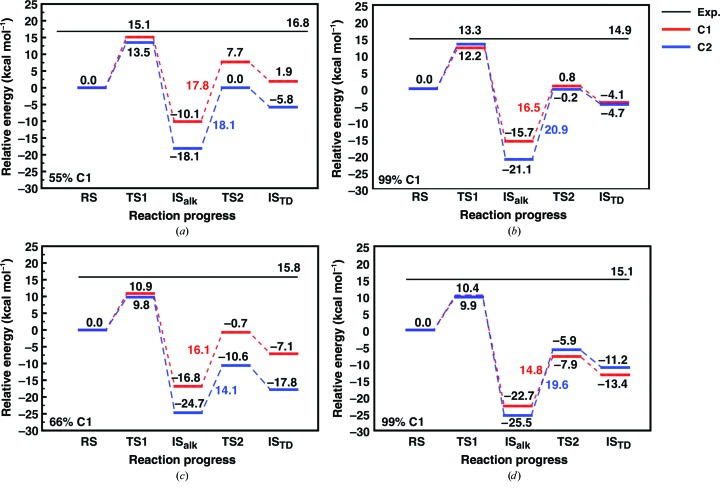
Calculated free-energy profiles for the StEH1-catalyzed hydrolysis of **1a** bound in the preferred of the two binding modes shown in Fig. 2 ▸. RS, TS1, IS_alk_, TS2 and IS_TD_ indicate the Michaelis complex, the transition state for nucleophilic attack on the epoxide, the alkylenzyme intermediate, the transition state for the hydrolysis of this intermediate and the resulting tetrahedral intermediate, respectively. All values are averages over 30 individual trajectories at each reacting state. Shown here are the reactions catalyzed by wild-type StEH1 (*a*, *b*) and R-C1 (*c*, *d*) for the (*R*,*R*)-enantiomer (*a*, *c*) and the (*S*,*S*)-enantiomer (*b*, *d*). The absolute energies for each reacting state are shown in black, and the relative energies for TS2 (relative to IS_alk_) are shown in red and blue for attack on C1 and C2, respectively. Finally, the experimentally observed regioselectivity for each system is shown at the bottom left of each plot (see Table 1 ▸). The corresponding raw data are shown in Table 3 ▸ and bar charts of the activation free energies are shown in Fig. 8 ▸.

The StEH1 variants tested here contain between two and six substitutions, depending on the variant, in residues putatively involved in substrate binding (Fig. 4 ▸, Table 2 ▸; Janfalk Carlsson *et al.*, 2012 ▸, 2016 ▸). The StEH1-catalyzed hydrolysis of (*R*,*R*)-**1a** results in a close-to-racemic mixture of the diol enantiomers (at the assay temperature used here, 30°C; Lindberg, de la Fuente Revenga *et al.*, 2010 ▸; regiopreference shown in Table 1 ▸). The EVB simulations similarly predict this low degree of regioselectivity with (*R*,*R*)-**1a**, with very similar barriers for hydrolysis of either the (*R*,*S*)- or (*S*,*R*)-alkylenzymes (C1 or C2; mode 1 in Table 3 ▸ and Figs. 7 ▸ *a* and 8 ▸ *a*). In addition, the calculated energy barriers suggest that in order to result in productive Michaelis complex(es), the substrate needs to be bound to StEH1 in mode 1 (Fig. 2 and Supplementary Fig. S5), in which the phenyl substituent points towards His300. This is the same preferred binding mode as has previously been suggested for the analogous StEH1-catalyzed hydrolysis of styrene oxide (Bauer *et al.*, 2016 ▸).

Our EVB calculations for the hydrolysis of (*R*,*R*)-**1a** suggest the following flux through the pathway modelled in Fig. 3 ▸. (i) Nucleophilic attack occurs preferentially at C2, but attack at C1 is also possible, albeit to a much lower degree. (ii) The barriers for hydrolysis of the alkylenzymes are equally high, which is in line with the observed product ratio (Figs. 7 ▸ and 8 ▸). The finding that the formation of one alkylenzyme is favoured over the other fits well with the experimental observations, where only one amplitude is detected although two product enantiomers are formed. Still, the formation of two product diols by necessity invokes the formation of an alkylenzyme after attack at C1 also, albeit at a lower, undetectable level. We note as an aside that as in our previous calculations of the hydrolysis of styrene oxide (Bauer *et al.*, 2016 ▸), as well as previous DFT calculations of epoxide hydrolase-catalyzed epoxide hydrolysis (Lind & Himo, 2016 ▸), we obtain unphysically exothermic reaction free energies for the initial ring opening (*i.e.* the alkylenzyme intermediate is far too exothermic compared with what would be expected from considering the experimental rates; see Table 1 ▸, which shows both *k*_2_ and *k*_−2_ where available). As discussed previously (Bauer *et al.*, 2016 ▸), we believe this to be a simulation artifact from the DFT calculations used to perform the fitting (see the Supporting Information). We have thus considered the energetics of the two reacting steps individually in all cases.

Following from this, the hydrolysis of (*S*,*S*)-**1a** yields only (*R*,*S*)-**2a**, suggesting that attack at C1 is favoured. The paradox here is that two distinct amplitudes are observed during the pre-steady state, with lifetimes that are described by two different observed rates (Lindberg *et al.*, 2008 ▸). Once again, our EVB calculations provide a plausible explanation for the contradictory observations of product ratios and the (postulated) presence of two alkylenzymes. The calculated energy barriers suggest that (*S*,*S*)-**1a** is also bound productively in mode 1 for the formation of alkylenzyme(s). Attack by the Asp105 carboxylate on the epoxide ring is possible at either C atom (Fig. 2 ▸ and Supplementary Fig. S6), with closely matched activation energies, thereby allowing the formation of either the pro-(1*R*,2*S*)-diol or pro-(1*S*,2*R*)-diol alkylenzyme (Table 3 ▸, Figs. 7 ▸ and 8 ▸). The energy barriers resulting from the simulation agree with the presence of two experimentally observed amplitudes in the pre-steady-state phase of the reactions. The barriers for the subsequent hydrolysis step, however, will only allow reaction of the pro-(1*R*,2*S*)-diol alkylenzyme; the activation energy for hydrolysis of the other alkylenzyme is >4 kcal mol^−1^ higher (Table 3 ▸, Figs. 7 ▸ and 8 ▸). This can explain the exclusive formation of the (1*R*,2*S*)-**2a** diol. Furthermore, the calculated barriers agree reasonably well with those back-calculated from determined microscopic rates, or *k*_cat_ in the case of (*R*,*R*)-**1a**, and also emphasize that hydrolysis is indeed the rate-determining step in the overall catalytic cycle with both enantiomers.

### 3.1. R-C1   

The two substitutions in the R-C1 variant (V141K and I155V) cause only small effects on enzyme activity expressed as either *k*_cat_ or *k*_cat_/*K*_m_, with the exception of the *k*_cat_ for (*R*,*R*)-**1a**, which is increased by fivefold (Table 1 ▸). As the *K*_m_ for (*R*,*R*)-**1a** is also elevated to a comparable extent, the overall enzyme efficiency is unaltered. This increase in turnover number may be owing to an elevated interconversion rate between the ES and E′S species (*k*_5_ + *k*_−5_ in Fig. 3 ▸), supported by the sixfold-increased value of the sum of these rates. The estimated value is similar to that previously determined for the wild-type enzyme at elevated temperatures (Lindberg, de la Fuente Revenga *et al.*, 2010 ▸). In the wild type, the value of this sum of flux rates is similar to the value of *k*_cat_ and may be rate-limiting at 30°C, but not at higher temperatures. The increase in the isomerization of the Michaelis complexes with this substrate may suggest that the mutations in R-C1 introduce some level of increased structural flexibility.

The activity with (*S*,*S*)-**1a** measured at the steady state is essentially unchanged. Effects from the substitutions can, however, be seen on the transient rates and on the dissociation constant of the Michaelis complex, *K*_S_ (Table 1 ▸). Similarly to wild-type StEH1, the transient alkylenzyme buildup and decay in the catalyzed hydrolysis of (*S*,*S*)-**1a** can only be modelled satisfactorily by a second-order exponential function. Hence, two distinct rates and amplitudes separated in time are recorded (Fig. 5 ▸ *a*). The faster observed rate (*k*_obs1_ in Fig. 6 ▸ *a*) follows a (‘normal’) hyperbolic dependence on substrate concentration and is modelled by (4), which describes rapid (pre-equilibrated) formation of the Michaelis complex, followed by alkyl­enzyme formation and decay. The determined values of the microscopic rates show an approximately fivefold increase in the alkylation rate (*k*_2_), while the decay rates (*k*_−2_ and *k*_3_) are essentially unchanged (Table 1 ▸). The estimated (*k*_5_ + *k*_−5_) rate from the concentration-dependence of the slower observed transient rates with (*S*,*S*)-**1a** (*k*_obs2_ in Fig. 6 ▸ *a*) is not significantly different from the corresponding wild-type value.

Structurally, we have considered the changes in the root-mean-square fluctuations (r.m.s.f.) of all backbone atoms during the equilibration of the Michaelis complexes for both possible binding modes of (*R*,*R*)-**1a** and (*S*,*S*)-**1a** in complex with the wild-type and R-C1 variant of StEH1 (Supplementary Fig. S2). We have also calculated the corresponding dynamic changes in active-site volume during these equilibration runs using the *POVME* 2.0 pocket-volume measurer (Durrant *et al.*, 2011 ▸, 2014 ▸; Table 4 ▸). From the r.m.s.f. plots, it can be seen that globally the backbone of the R-C1 variant appears to be more flexible than that of wild-type StEH1. The exception to this, however, appears to be in the regions around Tyr154 and in some instances Tyr235, where the incorporation of the I155V substitution appears to dampen the flexibility of these catalytically important tyrosine residues. We note that the overall flexibility trends mostly appear to be independent of the enantiomer or binding mode (*i.e.* the four plots are qualitatively similar). Tying in with this, the calculated active-site volumes shown in Table 4 ▸, as well as the corresponding standard deviations (which indicate how much the active-site volume changes over the course of the corresponding equilibration runs), show that for both enantiomers and binding modes of **1a** the active-site volume is more compact in the Michaelis complexes with the R-C1 variant than those with wild-type StEH1. We note, however, that these R-C1 volumes should be assessed also taking into account the flexibility of the Lys141 side chain, which is not present in the wild-type enzyme, and which will impact the *POVME* calculations based on whether the side chain is positioned in the active site or out of it in a given snapshot.

Similar to the wild type, the equal barriers for R-C1-catalyzed ring opening of (*S*,*S*)-**1a** contradict the fact that only (*R*,*S*)-**2a** is formed after its hydrolysis catalyzed by either the wild type or R-C1. However, as in the wild-type parent, the barrier for hydrolysing the intermediate formed after ring opening at C2 is 5 kcal mol^−1^ higher (Table 3 ▸). Thus, the stereoconfiguration of the diol product again becomes exclusively (1*R*,2*S*)-**2a**. The fact that the selectivity is determined at the hydrolysis step and not the alkylation step is in good agreement with our previous computational studies of the hydrolysis of other substrates by these enzymes (Amrein *et al.*, 2015 ▸; Bauer *et al.*, 2016 ▸). The calculated energy barriers for the hydrolysis of (*S*,*S*)-**1a** nicely explain the experimental observations of two transient amplitudes, indicative of two alkyl­enzymes, but resulting in only one product enantiomer. Also, although the magnitude is overestimated, the faster alkylation rate (*k*_2_ in Table 1 ▸) determined for R-C1 is caught by the calculations as lower barriers for ring opening compared with the wild type (Table 3 ▸).

The fluorescence-quenching signal recorded with all enzyme variants and with both enantiomers of **1b** could be fitted by a first-order exponential function in all cases. The catalytic activities of R-C1 are comparable to those of the wild-type enzyme, with the exception of the alkylation rate with (*S*,*S*)-**1b** which, similar to (*S*,*S*)-**1a**, is fivefold faster (Table 1 ▸). The dissociation constants with both these epoxides are also elevated to approximately the same degree, which may be connected to the increased alkylation rates.

### 3.2. R-C1B1   

The R-C1B1 variant contains two additional substitutions: W106L and L109Y. Trp106 is a relatively conserved residue in epoxide hydrolases of the α/β-hydrolase family (The Epoxide Hydrolase Database; http://www.led.uni-stuttgart.de; Barth *et al.*, 2004 ▸) and contributes its main-chain amide to the oxyanion hole, stabilizing the tetrahedral intermediate formed during the hydrolytic decomposition of the alkylenzyme (Fig. 1 ▸ *c*). The indole of this residue has also been suggested to contribute favourably to the binding of aryl-substituted epoxides such as **1b** and styrene oxide (Amrein *et al.*, 2015 ▸; Bauer *et al.*, 2016 ▸). The second substitution, L109Y, spatially located adjacent to Trp106, does not reintroduce an aromatic ring to the substrate-binding site since the phenolic side chain swings out of the active site and engages in a hydrogen bond to the side chain of Asn241 (Fig. 4 ▸ *b*).

The structural changes in this variant cause relatively larger effects on the catalytic rates (Table 1 ▸), with a 30-fold decrease in *k*_cat_/*K*_m_ with (*R*,*R*)-**1a** compared with R-C1. The decrease in catalytic activity is primarily manifested owing to a lower turnover number (*k*_cat_) when compared with R-C1. Owing to the absence of a clear dependence of the observed rates (*k*_obs_) on substrate concentration, we have been unable to determine the individual rate constants under multiple-turnover conditions for the R-C1B1-catalyzed hydrolysis of either (*R*,*R*)-epoxide. This can occur if two different alkylenzymes are produced on the same timescale. Therefore, we did not attempt to perform EVB calculations for this or the following variants, as the missing information about the individual rates would make the comparison between simulation and experiment meaningless. Under such circumstances the different signal amplitudes can mix, with the observable result of an apparent overall lack of substrate-concentration dependence. The inset in Fig. 6 ▸(*c*) shows the behaviour of the observed transient rates as a function of (*R*,*R*)-**1a** concentration. A possible increase in the observed rates may be present at higher substrate concentrations, rather than the decrease seen for the wild-type and R-C1 enzymes. The activity with (*S*,*S*)-**1a** was less affected, with a mere fourfold decrease in *k*_cat_/*K*_m_ compared with R-C1. There was no indication of two observed transient rates (Fig. 5 ▸ *b*), suggesting a rapid equilibrium between the two Michaelis complexes. The most notable effect on the kinetics is an approximately 20-fold decrease in the decomposition rate from the alkylenzyme back to the Michaelis complex (*k*_−2_). Since the hydrolysis rate is also slower compared with the wild type and R-C1, this is expected to stabilize the alkylenzyme to a greater degree, which may be the cause of the lower hydrolysis rate (and *k*_cat_).

The corresponding rates with (*S*,*S*)-**1b** were also altered; the alkylation rate was almost 15-fold lower compared with R-C1, while the rates of alkylenzyme decay were similar for the two variants. This lower alkylation rate explains the tenfold increase in the *K*_m_ of (*S*,*S*)-**1b** in this variant (see the expression for the *K*_m_ term in the denominator of equation 6), and also identifies the alkylation step to be partially rate-limiting in this case.

The relatively tight binding of the **1b** enantiomers to these enzymes made single-turnover experiments possible, which allows the direct extraction of the hydrolysis rates, *k*_3_. With (*S*,*S*)-**1b**, the observed single-turnover rates were close to the cognate *k*_cat_ values (Table 1 ▸), underlining that hydrolysis is indeed rate-determining in these reactions. On the other hand, for wild-type- and R-C1-catalyzed hydrolysis of (*R*,*R*)-**1b** the determined hydrolysis rates were substantially faster than *k*_cat_, suggesting that another, yet unidentified, step on the reaction pathway may be rate-limiting and contributing to the value of *k*_cat_. The alkylation rates (*k*_2_ in Table 1 ▸) clearly showed that this step is *not* rate-limiting for *k*_cat_, which is corroborated by the calculated barriers of the wild-type reaction (Amrein *et al.*, 2015 ▸). Hence, a step downstream of the hydrolysis step is suspected to influence *k*_cat_ in these reactions.

### 3.3. R-C1B1D33   

This variant has picked up one additional substitution: F189L (Fig. 4 ▸). The replacement has not caused any major effects on the catalytic efficiencies with either epoxide **1a** or **1b** compared with its parent R-C1B1 (Table 1 ▸). There are, however, some noteworthy changes: *k*_cat_ with either (*S*,*S*)-epoxide is increased. The observed rates of alkylenzyme formation and decay during the pre-steady-state phase could, as in the case of R-C1B1, be satisfactorily modelled by a single exponential. The estimated microscopic rates with (*S*,*S*)-**1a** point towards a lower energy barrier for alkylenzyme formation coupled with a decreased affinity for the substrate [*K*_S_^(*S*,*S*)-**1a**^ is >6 m*M*]. This suggests that the inserted substitution allows a higher degree of flexibility of the Michaelis complex, possibly facilitating productive binding modes for nucleophilic attack. The microscopic rates with (*S*,*S*)-**1b** were increased to values that escaped detection outside the dead time of the stopped-flow equipment.

### 3.4. R-C1B1D33E6   

This final, fourth-generation variant contains an additional L266G substitution (Fig. 4 ▸). This mutation is detrimental to the activity with either of the (*R*,*R*)-enantiomers tested here (Table 1 ▸). The activities with the (*S*,*S*)-epoxides are also lower (20–60-fold with **1a** and **1b**, respectively). The steady-state parameters with (*S*,*S*)-**1b** indicate that both turnover and *K*_m_ have been affected. As discussed in Janfalk Carlsson *et al.* (2016 ▸), this variant has the largest active-site cavity: ∼250 Å^3^ larger than its parent. This larger volume may allow new binding modes that are favourable for the initial binding step but unfavourable for the subsequent formation of the alkyl­enzyme. We note that this variant was originally isolated for its ability to transform benzyloxirane into (2*R*)-3-phenylpropane-1,2-diol; hence, the enantiomeric purity of the product was prioritized over catalytic efficiency in this case (Janfalk Carlsson *et al.*, 2012 ▸).

## 4. Conclusions   

It has been established that the epoxide ring opening and hydrolysis catalyzed by StEH1 proceeds *via* an S_N_2 mechanism, causing inversion of configuration at the attacked epoxide C atom (Lindberg *et al.*, 2008 ▸; Janfalk Carlsson *et al.*, 2012 ▸). A mixture of diol products after hydrolysis therefore requires the presence of branched reaction pathways. We show here for the first time that flux through such complex reaction pathways is affected by kinetic rates between noncovalent Michaelis complexes, the rates of formation of the respective covalent intermediates and the rates of their subsequent hydrolyses. Specifically, by employing a series of related StEH1 variants, evolved *in vitro*, we have been able to link structural effects, in particular changes in the active-site cavity as well as preferred binding modes across different variants, to stepwise changes in catalytic function. Providing a molecular understanding of how such subtle structural effects can manipulate complex kinetic pathways is a major advance towards the efficient generation of novel enzymes with tailored enantioselectivities and regioselectivities.

## Supplementary Material

Details of computational methodology, supplementary figures and tables.. DOI: 10.1107/S2052252518003573/jt5024sup1.pdf

## Figures and Tables

**Figure 8:**
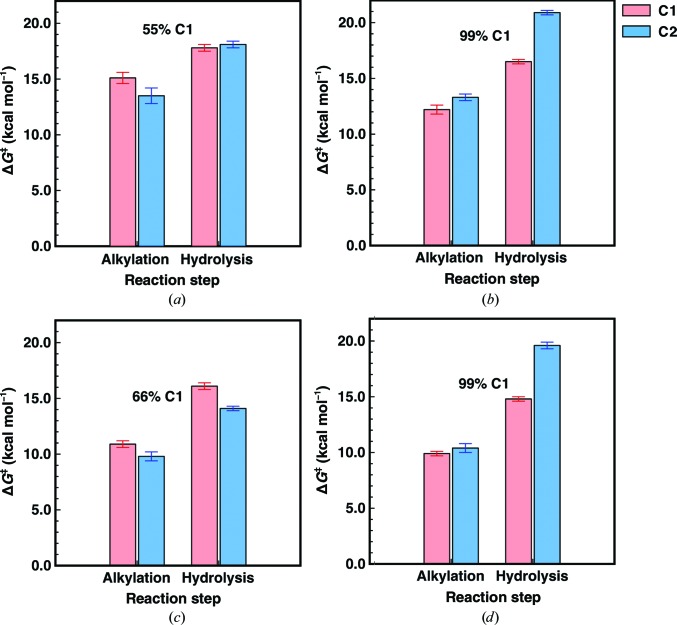
Calculated activation free energies for the alkylation and hydrolysis steps in the StEH1-catalyzed hydrolysis of **1a** bound in the preferred of the two binding modes shown in Fig. 2 ▸. All values are averages and standard errors of the mean (shown as error bars) over 30 individual trajectories. Shown here are the reactions catalyzed by wild-type StEH1 (*a*, *b*) and R-C1 (*c*, *d*) for the (*R*,*R*)-enantiomer (*a*, *c*) and the (*S*,*S*)-enantiomer (*b*, *d*). The experimentally observed regioselectivies are indicated for each system (see Table 1 ▸). The corresponding raw data are shown in Table 3 ▸ and the full free-energy profiles for the preferred binding modes are shown in Fig. 7 ▸.

**Table 1:** Kinetic parameters and regioselectivity in the hydrolysis of **1a** and **1b**

	*k*_cat_ (s^−1^)	*K*_m_ (µ*M*)	*k*_cat_/*K*_m_ (s^−1^ m*M* ^−1^)	*K*_S_ (µ*M*)	*k*_2_ (s^−1^)	*k*_−2_ (s^−1^)	*k*_3_ (s^−1^)	*k*_5_ + *k*_−5_ (s^−1^)	Regioselectivity
(*R*,*R*)-**1a**									
WT	4.7 ± 0.7†	490 ± 100†	9.7 ± 2†	—	—	—	—	8.1 ± 3†	55% C1
R-C1	25 ± 4‡	3300 ± 10‡	7.7 ± 0.2‡	—	—	—	—	48 ± 70	66% C1
R-C1B1	0.92 ± 0.4‡	3400 ± 200‡	0.26 ± 0.01‡	—	—	—	—	—	89% C2
R-C1B1D33	Not saturating	Not saturating	0.25 ± 0.01‡	—	—	—	—	—	91% C2
R-C1B1D33E6	—	—	<0.008‡	—	—	—	—	—	—
(*S*,*S*)-**1a**									
WT	63 ± 3†	77 ± 10†	820 ± 100†	470 ± 100†	370 ± 20†	170 ± 20† §	110 ± 10† §	32 ± 2†	99% C1
R-C1	65 ± 2‡	110 ± 10‡	560 ± 40‡	1600 ± 900	2100 ± 600	240 ± 60§	75 ± 30§	44 ± 50	99% C1
R-C1B1	31 ± 1‡	240 ± 20‡	130 ± 6‡	2700 ± 2000	1400 ± 800	10 ± 30§	32 ± 20§	—	99% C1
R-C1B1D33	79 ± 5‡	410 ± 40‡	190 ± 20‡	6500 ± 6000	2600 ± 2000	74 ± 90§	84 ± 90§	—	99% C1
R-C1B1D33E6	Not saturating	Not saturating	10 ± 0.2‡	—	—	—	—	—	99% C1
(*R*,*R*)-**1b**									
WT	17 ± 0.5	16 ± 1	1100 ± 50	7.8 ± 13	180 ± 100	13 ± 10	42 ± 0.2¶	—	Meso product
R-C1	12 ± 0.5	20 ± 2	610 ± 40	13 ± 17	85 ± 3	22 ± 40	28 ± 0.2¶	—	Meso product
R-C1B1	0.51 ± 0.09	170 ± 40	3.1 ± 0.1	—	—	—	—	—	Meso product
R-C1B1D33	0.38 ± 0.09	190 ± 60	2.0 ± 0.1	—	—	—	—	—	Meso product
R-C1B1D33E6	—	—	<0.1	—	—	—	—	—	Meso product
(*S*,*S*)-**1b**									
WT	4.0 ± 0.06	1.0 ± 0.08	3900 ± 200	5.5 ± 0.9	23 ± 0.7	4.5 ± 0.8	3.9 ± 0.005¶	—	Meso product
R-C1	4.4 ± 0.09	1.3 ± 0.09	3300 ± 20	16 ± 5	120 ± 9	4.2 ± 6	5.4 ± 0.01¶	—	Meso product
R-C1B1	4.0 ± 0.09	16 ± 0.9	250 ± 0.09	21 ± 10	8.9 ± 1	5.1 ± 0.08	3.0 ± 0.01¶	—	Meso product
R-C1B1D33	12 ± 0.7	65 ± 6	180 ± 6	—	Too fast	—	—	—	Meso product
R-C1B1D33E6	0.42 ± 0.09	110 ± 30	3.1 ± 0.2	—	Too fast	—	—	—	Meso product

† Data from Lindberg, Ahmad *et al.* (2010 ▸).

‡ Data from Janfalk Carlsson *et al.* (2012 ▸).

§ Calculated from the steady-state expression for *k*_cat_ and the determined values of *k*_cat_ and (*k*_−2_ + *k*_3_).

¶ Experimentally determined in a single-turnover experiment.

**Table 2:** Amino-acid sequences of StEH1 variants

	Sequence position					
Enzyme	106	109	141	155	189	266
Wild type	Trp	Leu	Val	Ile	Phe	Leu
R-C1	Trp	Leu	Lys	Val	Phe	Leu
R-C1B1	Leu	Tyr	Lys	Val	Phe	Leu
R-C1B1D33	Leu	Tyr	Lys	Val	Leu	Leu
R-C1B1D33E6	Leu	Tyr	Lys	Val	Leu	Gly

**Table 3:** Experimental and calculated energy barriers for hydrolysis of the different enantiomers of **1a** by either wild-type StEH1 or the R-C1 variant Δ*G* ^‡^ and Δ*G*° denote activation and reaction free energies, respectively, for either the formation of the alkylenzyme intermediate (Δ*G* ^‡1^ and Δ*G*°^1^) or the hydrolysis of this intermediate (Δ*G* ^‡2^ and Δ*G*°^2^) (note that Δ*G* ^‡2^ and Δ*G*°^2^ are the energies relative to the alkylenzyme intermediate, rather than relative to the Michaelis complex; the absolute values relative to the Michaelis complex along the reaction profile are shown in Fig. 7 ▸). All energies are provided in kcal mol^−1^ and are given as averages and standard error of the mean over 30 individual trajectories. The data for the preferred mode for each system are shown in Figs. 7 ▸ and 8 ▸.

	Δ*G* ^‡1^	Δ*G*°^1^	Δ*G* ^‡1^ (exp)†	Δ*G* ^‡2^	Δ*G*°^2^	Δ*G* ^‡2^ (exp)
Wild-type StEH1						
(*R*,*R*)-**1a**						
C1, mode 1	15.1 ± 0.5	−10.1 ± 0.5	No available data	17.8 ± 0.3	12.0 ± 0.4	16.8‡
C1, mode 2	17.7 ± 0.4	−6.5 ± 0.6	No available data	19.8 ± 0.3	13.5 ± 0.4	16.8‡
C2, mode 1	13.5 ± 0.7	−18.1 ± 0.7	No available data	18.1 ± 0.3	12.3 ± 0.4	16.8‡
C2, mode 2	17.3 ± 0.3	−5.5 ± 0.5	No available data	24.2 ± 0.2	18.7 ± 0.3	16.8‡
(*S*,*S*)-**1a**						
C1, mode 1	12.2 ± 0.4	−15.7 ± 0.6	14.2§	16.5 ± 0.2	11.6 ± 0.3	14.9¶
C1, mode 2	16.8 ± 0.5	−8.0 ± 0.8	14.2§	18.7 ± 0.3	13.6 ± 0.3	14.9¶
C2, mode 1	13.3 ± 0.3	−21.1 ± 0.4	14.2§	20.9 ± 0.2	16.4 ± 0.3	14.9¶
C2, mode 2	13.8 ± 0.4	−13.0 ± 0.6	14.2§	25.4 ± 0.2	20.4 ± 0.2	14.9¶
R-C1						
(*R*,*R*)-**1a**						
C1, mode 1	10.9 ± 0.3	−16.8 ± 0.5	No available data	16.1 ± 0.3	9.7 ± 0.4	15.8‡
C1, mode 2	16.3 ± 0.3	−8.7 ± 0.5	No available data	14.3 ± 0.2	6.6 ± 0.3	15.8‡
C2, mode 1	9.8 ± 0.4	−24.7 ± 0.7	No available data	14.1 ± 0.2	6.9 ± 0.3	15.8‡
C2, mode 2	15.5 ± 0.4	−7.6 ± 0.4	No available data	21.6 ± 0.2	15.2 ± 0.2	15.8‡
(*S*,*S*)-**1a**						
C1, mode 1	9.9 ± 0.2	−22.7 ± 0.3	13.1§	14.8 ± 0.2	9.3 ± 0.3	15.1¶
C1, mode 2	16.0 ± 0.6	−10.5 ± 0.7	13.1§	18.0 ± 0.2	11.9 ± 0.3	15.1¶
C2, mode 1	10.4 ± 0.4	−25.5 ± 0.5	13.1§	19.6 ± 0.3	14.3 ± 0.4	15.1¶
C2, mode 2	12.5 ± 0.6	−15.4 ± 0.8	13.1§	24.7 ± 0.2	20.0 ± 0.2	15.1¶

† Calculated from the experimentally determined rates in Table 1 ▸.

‡ Calculated from *k*_cat_.

§ Calculated from *k*_2_.

¶ Calculated from *k*_3_.

**Table 4:** Active-site volume analysis using *POcket Volume MEasurer* (*POVME*; Durrant *et al.*, 2011 ▸, 2014 ▸) Shown here are the average active-site volumes, and corresponding standard deviations, from our equilibration runs for the Michaelis complexes of wild-type and R-C1 StEH1 in complex with (*R*,*R*)-**1a** and (*S*,*S*)-**1a**, respectively. Both binding modes considered in this work (Fig. 2 ▸) are shown in this table. To generate these data, the centre of the search sphere for the volume was set in the active-site cavity, close to the catalytic Asp105, and kept constant for all calculations for direct comparability. The total volume search radius was set to 10 Å starting from this point, with a grid spacing of 0.5 Å. The second search sphere was placed further away in the active site to make sure that only grid points actually connected to the central active-site cavity were considered when calculating the active-site volume. A point was only considered to be connected if at least four other points were connected to the central sphere, and only grid points further away than 1.09 Å from a protein atom were counted towards the available surface volume. The calculations were performed on the 300 ns equilibration trajectories after stripping out both water atoms and the substrate molecule. This leads to data collection over 6000 data points for each of the systems considered here (*i.e.* a snapshot was taken every 50 ps of equilibration per trajectory), with the final volumes (Å^3^) presented as averages and standard deviations over all individual structures.

	Mode 1	Mode 2
(*R*,*R*)-**1a**		
Wild type	201.7 ± 21.2	190.2 ± 16.3
R-C1	146.2 ± 29.2	163.3 ± 18.9
(*S*,*S*)-**1a**		
Wild type	192.7 ± 16.1	182.5 ± 16.3
R-C1	151.6 ± 31.8	156.7 ± 19.4
