# Supplementary material for: Epoxide hydrolysis as a model system for understanding flux through a branched reaction scheme
Source: IUCrJ. 2018 Mar 22;5(Pt 3):269–82. doi: 10.1107/S2052252518003573 (PMC5929373; doi:10.1107/S2052252518003573)
Supplement: Supplementary file 1 [file m-05-00269-sup1.pdf]

IUCrJ

Volume 5 (2018)

Supporting information for article:

Epoxide Hydrolysis as a Model System for Understanding Flux Through a Branched Reaction Scheme

Åsa Janfalk Carlsson, Paul Bauer, Doreen Dobritzsch, Shina C. L. Kamerlin and Mikael Widersten

Supplementary Information for:

Epoxide Hydrolysis as a Model System for Understanding Flux Through a Branched Reaction Scheme

Åsa Janfalk Carlsson[†], Paul Bauer[‡], Doreen Dobritzsch[†], Shina C. L. Kamerlin[‡,*] and Mikael Widersten[†,*]

[†] Department of Chemistry – BMC, Uppsala University, Box 576, S-751 23 Uppsala, Sweden.

[‡] Science for Life Laboratory, Department of Cell and Molecular Biology, Uppsala University, BMC Box 596, S-751 24 Uppsala, Sweden

Corresponding Author Email Addresses:

mikael.widersten@kemi.uu.se and kamerlin@icm.uu.se

Table of Contents

S1. Simulation Details…………………………………………………………………......S3

S2. Supplementary Scheme………………………………………………………...…..S8

Scheme 1. Model of kinetic mechanism for epoxide hydrolysis by StEH1……………………..S8

S3. Supplementary Figures……………………………………………………..………...S9

Figure S1. Valence bond states used to describe the StEH1-catalyzed hydrolysis of **1a**…….....S9

Figure S2. Root mean square fluctuations of all StEH1 backbone C_α atoms during our initial molecular dynamics simulations …...……………………………………………………..S10

Figure S3. Root mean square deviations of all StEH1 backbone C_α atoms during our initial molecular dynamics simulations …...……………………………………………………..S11

Figure S4. Root mean square deviations of all substrate atoms during our initial molecular dynamics simulations……………………………………………………………...S12

Figure S5. Key stationary points for the hydrolysis of (*R*,*R*)-**1a** by wild-type StEH1 ……….S13

Figure S6. Key stationary points for the hydrolysis of (*S*,*S*)-**1a** by wild-type StEH1 …..…….S14

S4. Supplementary Tables…………………………………………..………………..S15

Table S1. Calculated energetics for the uncatalyzed hydrolysis of both enantiomers of **1a**…..S15

Table S2. Absolute quantum chemical energies for the uncatalyzed hydrolysis of **1a**………..S16

Table S3. Distances between key atoms during the hydrolysis of **1a** by StEH1.......................S17

S5. Empirical Valence Bond Parameters……………………………...…………..S18

Figure S7. Atom numbering to describe the reacting system in our EVB calculations……….S18

Table S4. EVB parameters used in this work………………………………………………S19

Table S5. Van der Waals parameters used to describe the reacting system…………………...S19

Table S6. Atom types in different VB states used to describe the reacting system…………...S20

Table S7. Partial charges of each atom used to describe the reacting system……………….....S21

Table S8. Bond parameters for covalent bonds used to describe the reacting system………...S23

Table S9. Bond types in different VB states used to describe the reacting system…………....S24

Table S10. Angle parameters used for bending adjacent bonds in the reacting system……….S26

Table S11. Angle types of the different VB states used to describe the reacting system……...S27

Table S12. Torsion parameters used to describe the reacting system………………………..…...S30
Table S13. Torsion types in the different VB states used to describe the reacting system…......S31
Table S14. Improper torsion parameters used to describe the reacting system………………..S34
Table S15. Improper torsion angles of the VB states used to describe the reacting system…...S35

S6. Cartesian Coordinates for Key Stationary Points…………………………..………..S36

S7. References……………………………………………………………………..………..S53

S1. Simulation Details

Following from our previous work on the StEH1-catalyzed hydrolysis of styrene oxide and *trans*-stilbene oxide,[1,2] we have used the empirical valence bond (EVB) approach[3] to model the chemical step for the hydrolysis of **1a**. The substrate and key intermediates were parameterized as in our previous work, using the OPLS-AA force field,[4] version 11, as implemented in Macromodel *v.* 9.1.[5] Partial charges were obtained using the restrained electrostatic potential (RESP) approach,[6] by calculating the electrostatic potential at the HF/6-31G* level of theory, using Gaussian 09 Rev. C.01,[7] and fitting the potential to the atomic coordinates using ANTECHAMBER.[8] All EVB parameters used in this work are presented in as **Supplementary Tables** below. All molecular dynamics (MD) and EVB simulations were performed using the OPLS-AA force field[3] and the Q simulation package.[9] All energetic analysis was performed using the Qfep and Qcalc modules of Q, and all geometric analysis was performed by post-processing of the Q trajectories using GROMACS v. 5.0.5.[10]

Coordinates for the wild-type enzyme and the C1 variant were taken from the Protein Data Bank,[11] PDB IDs 2cjp[12] and 4uhb[13] respectively. We note that the K141 side chain (V141K substitution from wild-type) is poorly resolved in the C1 structure; however, as this is a solvent exposed lysine that is likely to be highly flexible, we kept the crystallographic coordinates in our simulations. As in our previous work,[1] both enantiomers of **1a** were manually placed in the active site in such a way as to optimize the angle of attack for the nucleophile, protein-substrate interactions, and prevent unbinding of the substrate during the simulations. Each enantiomer was placed in each of the two binding modes shown in **Figure 2** of the main text, leading to a total of four independent enantiomer/binding mode combinations per enzyme variant.

The structures were prepared for the EVB simulations as in our previous studies.[1,2] In brief, the system was solvated by a 20Å droplet of TIP3P water molecules,[14] centered on the γ-carbon of

D105, and with the bonds to hydrogen atoms restrained using the SHAKE algorithm.[15] This droplet was then described using a multi-layer model, in the framework of the surface constrained all atom solvent approach (SCAAS) as implemented in Q v. 5.0,[9] in which all atoms in the inner 85% of the sphere (17Å from the sphere center) were unrestrained and fully flexible during our simulations, all atoms in the outer 15% of the sphere (outer 3Å layer) were subjected to 20 kcal mol^{-1} $Å^{-2}$ harmonic restraint to reduce their mobility, and all atoms outside the explicit sphere were restrained to their crystallographic positions using a 200 kcal mol^{-1} $Å^{-2}$ harmonic restraint. Tying in with this, all amino acid side chains within the mobile region, *i.e.* the inner 17Å of the sphere, were ionized according to the most likely ionization state predicted by PROPKA (v. 3.0),[16,17] and all residues located outside this sphere were kept in their neutral form to avoid unsolvated charges from exerting unphysical electrostatic effects on the reacting region, in line with our previous work.[1,18,19] The most likely histidine protonation states were predicted using the MOLPROBITY server,[20] and H104 was kept protonated, in line with our previous work.[1,2] This led to an overall system charge of -1 for both variants. For a list of residues ionized during the simulations, and histidine protonation states, see the supporting information of ref. 2.

Once the structures had been prepared for the simulations, each system (at the Michaelis complex) was first minimized at close to 0K with an extremely small step size to ease initial bad contacts, after which the step size was set to 1 fs for the remainder of the simulation, with a final MD minimization at 1K for 30 ps. The system was equilibrated by gradually heating the water sphere for 30 ps at 100K and another 30 ps at 300K, while keeping all solute atoms (including the catalytic water and substrate) restrained to their crystallographic positions with a 200 kcal mol^{-1} $Å^{-2}$ harmonic restraint. The system was then cooled down again to 5K, and then gradually reheated to first 30, then 150, then 300K, while dropping the restraints from 200 kcal mol^{-1} $Å^{-2}$ on all solute atoms to 200 kcal mol^{-1} $Å^{-2}$ on only the reacting atoms (**Figure S1**). Once this heating-

cooling-heating procedure was complete, the system was then equilibrated for a further 10 ns as preparation for our EVB simulations, and the root mean square deviations (RMSD) of all C_α atoms during out simulations are shown in **Figure S3** for the wild-type and R-C1 variant respectively, and the corresponding values for the substrate are shown in **Figure S4**. A 1 fs step size was used throughout, and temperature was kept constant using Berendsen's thermostat[21] with a bath coupling of 100 fs. During the whole procedure, the non-bonded pair lists were updated every 30 ps, with a cutoff for the explicit interactions at 10 Å, while long range electrostatic interactions beyond this distance were modelled using the Local Reaction Field (LRF)[22] approach without any cutoff. The interactions of the atoms in the reacting region had no cutoff assigned to them and explicitly interacted with the complete system. This procedure was repeated 30 times per system, using different initial velocities, to a total simulation time of 300 ns per system and 2.4 µs over all systems. In each case, the final equilibrated structure was then used as the starting point for 30 independent EVB simulations per system.

As in our previous work, all EVB calculations were performed in a two-step process, with the reacting system described using the valence bond states shown in **Figure S1**. The EVB free energy perturbation/umbrella sampling (FEP/US) calculations were performed in 51 individual mapping windows of 200 ps in length each, to a total simulation time of 10.2 ns per system and 306 ns per reacting system/trajectory (4.9 µs over all trajectories). The EVB simulations were performed in two sequential steps, simulating first nucleophilic attack on the epoxide ring to yield the alkylenzyme intermediate, followed by water attack on the alkylenzyme intermediate to yield the transition state for the formation of the tetrahedral intermediate, using the end-points of the first simulation step as starting points for the second simulation step (see the main text for the reaction mechanism). From this point on, the velocities of all atoms in the systems were regenerated according to the Maxwell distribution at 300 K, before letting the system follow the

path towards both the alkylenzyme and tetrahedral intermediates again, using the same amount of sampling as for the first reaction step. This was necessary to obtain the correct alignment of the nucleophilic water molecule for proton abstraction by H300 during the formation of the tetrahedral intermediate. System energies were saved every 10 fs, and used to generate the EVB free energy profiles.

The corresponding off-diagonal and gas-phase shift parameters necessary to generate the EVB free energy profiles for each system are presented in **Section S5** (for details of what these parameters are, see for example reviews in refs. 23 and 24. These parameters were obtained by modeling the corresponding uncatalyzed reaction in aqueous solution using the substrate, nucleophilic water molecule, methyl imidazole and propionate (as models for the D105/H300 side chains, respectively), and fitting the free energy profiles for the corresponding uncatalyzed reaction to results obtained from quantum chemical calculations. The quantum chemical calculations were performed using Gaussian 09, revision E.01, with the system described using the ωB97X-D functional. Stationary points were optimized using the 6-31+G* basis set and impicit solvation using the SMD method, while frequency calculations were performed at the 6-311+G** level of theory. The intrinsic reaction coordinates were followed from the transition state to the respective reactant and product states, with the final energies calculated for the fragments at infinite separation. All pathways were calculated individually, with the lowest energy one then being used to parametrize the respective reference reactions.

For the quantum calculations, the transition states obtained from the EVB calculations were further optimized using the functional mentioned above towards the stationary points. From this point, the internal reaction coordinates (IRC)[25,26] were followed both towards the reactant and product states to obtain the QM potential energy surface for the first reaction step of the enzymatic mechanism. The final points of the IRC were again optimized to the stationary points,

together with the reactants at infinite separation. The resulting energetics were used for fitting the EVB parameters in aqueous solution for each system (attack at C-1 and C-2 for each enantiomer; note that the two binding modes are expected to have the same energy in aqueous solution as the environment is homogenous). The corresponding results are presented in **Table S1**. All parameters were then transferred *unchanged* to the corresponding enzymatic system, which is made feasible by the phase-independence of the EVB off-diagonal term as discussed in detail in for example ref. 27.

Finally, **Figures S5** and **S6** show the structures of key stationary points for the hydrolysis of (*R*,*R*)- and (*S*,*S*)-**1a** catalyzed by wild-type StEH1 respectively, and the corresponding geometric parameters are shown in **Table S2**.

S2. Supplementary Scheme

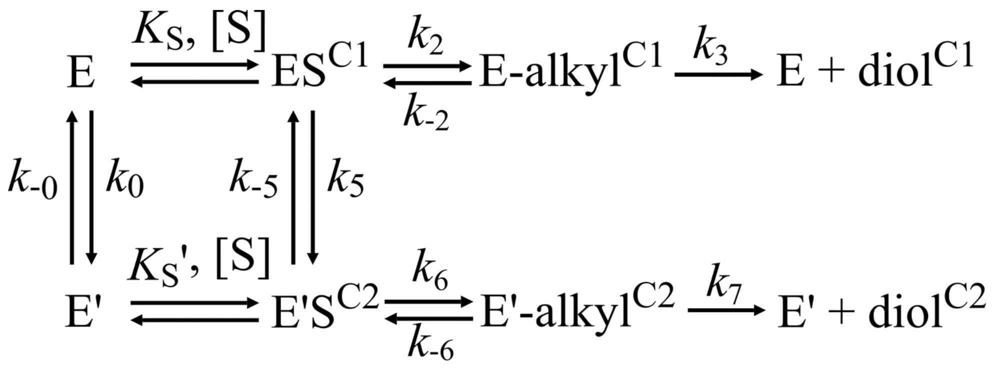

Scheme S1. Model of kinetic mechanism for epoxide hydrolysis catalyzed by StEH1. The model is based on data from analysis of pre-steady state and steady state kinetics as well as the enantiomeric ratios of the product diols.

S3. Supplementary Figures

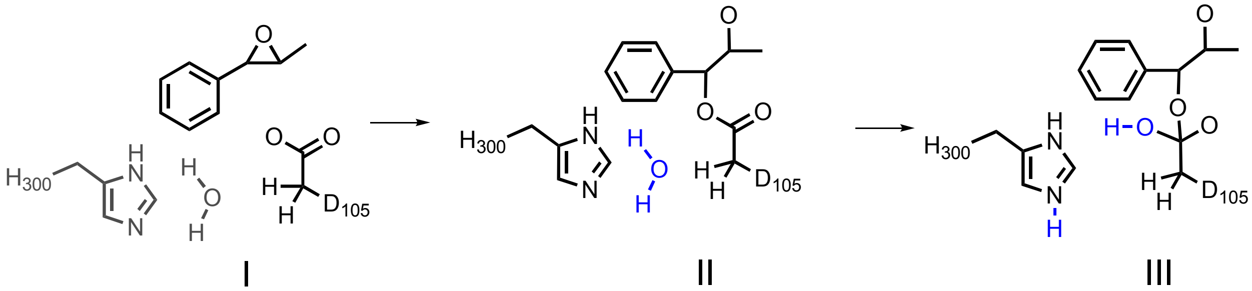

Figure S1: Valence bond states used to describe the StEH1-catalyzed hydrolysis of **1a** in our empirical valence bond calculations (for the corresponding EVB parameters, see **Section S5**). These states correspond to the Michaelis complex (I), the alkylenzyme intermediate (II) and the tetrahedral intermediate (III). The nucleophilic water molecule is highlighted in blue in States II and III, to show the position of the proton being transferred to H300.

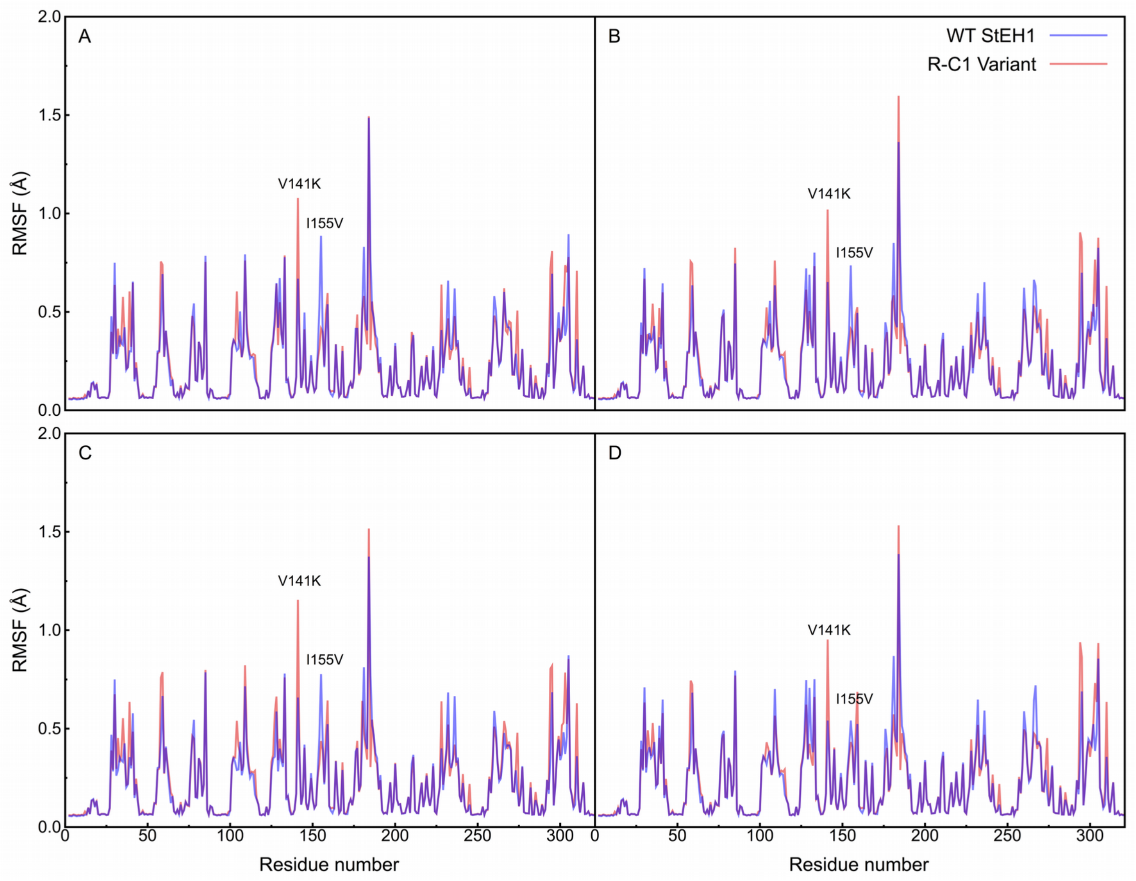

Figure S2: Overlay of the root mean square fluctuations (RMSF, Å) of all StEH1 backbone atoms during our initial 30 ns molecular dynamics simulations of the wild-type and R-C1 variants of StEH1, in complex with (A, B) (*R*,*R*)- and (C, D) (*S*,*S*)-**1a**, at the respective Michaelis complexes. Shown here are two enantiomers in binding modes 1 and 2 (left and right panels respectively, for a definition of the two different binding modes, see **Figure 2** of the main text).

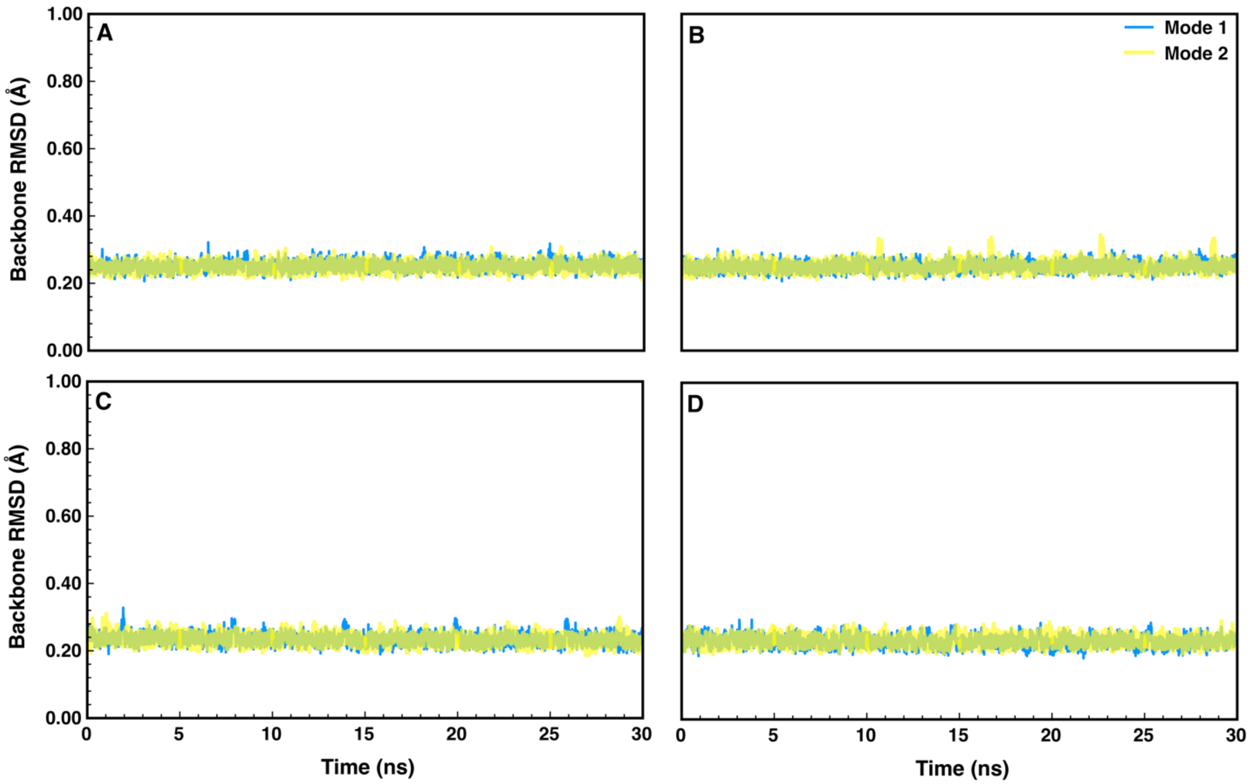

Figure S3: Root mean square deviations (RMSD, Å) of all StEH1 backbone Cα atoms during our initial 30 ns molecular dynamics simulations of the (A,B) wild-type and (C,D) R-C1 variants of StEH1, in complex with (A,C) (*R*,*R*)- and (B,D) (*S*,*S*)-1a. All values are averages over three independent trajectories.

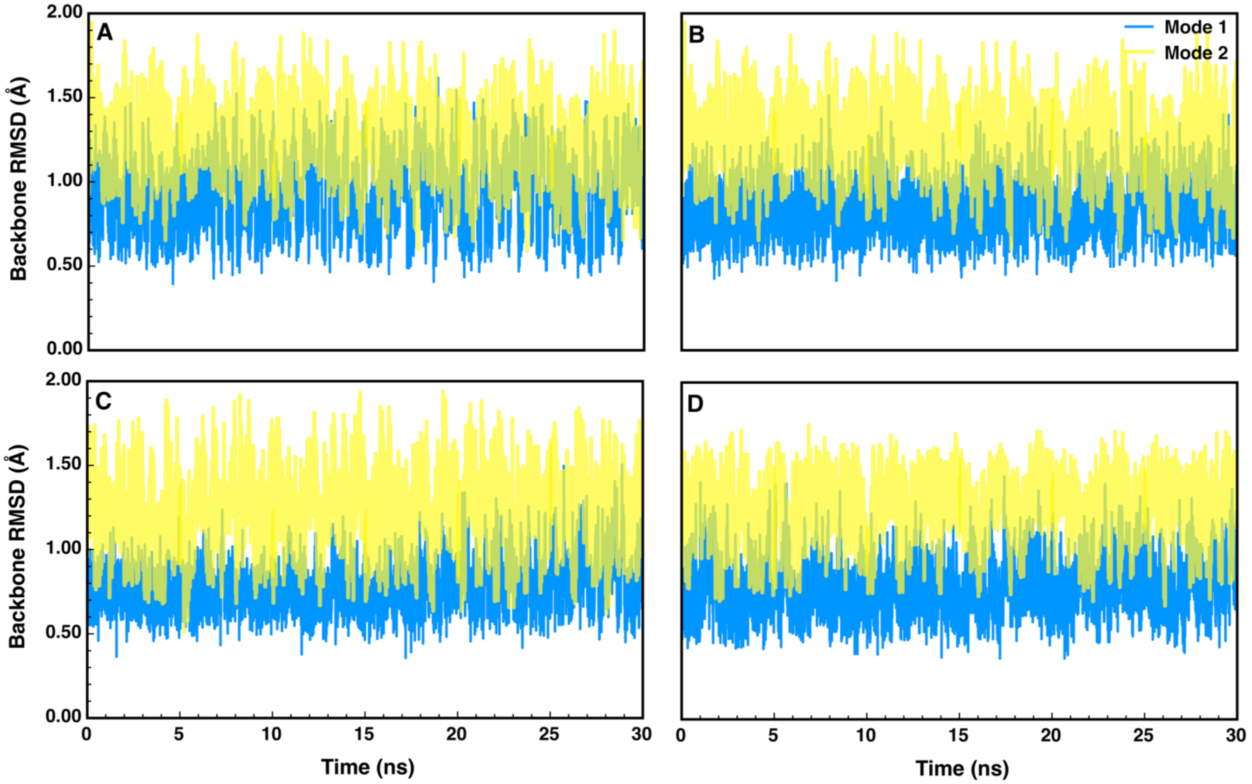

Figure S4: Root mean square deviations (RMSD, Å) of all substrate atoms during our initial 30 ns molecular dynamics simulations of the (A,B) wild-type and (C,D) R-C1 variants of StEH1, in complex with (A,C) (*R*,*R*)- and (B,D) (*S*,*S*)-**1a.** All values are averages over three independent trajectories.

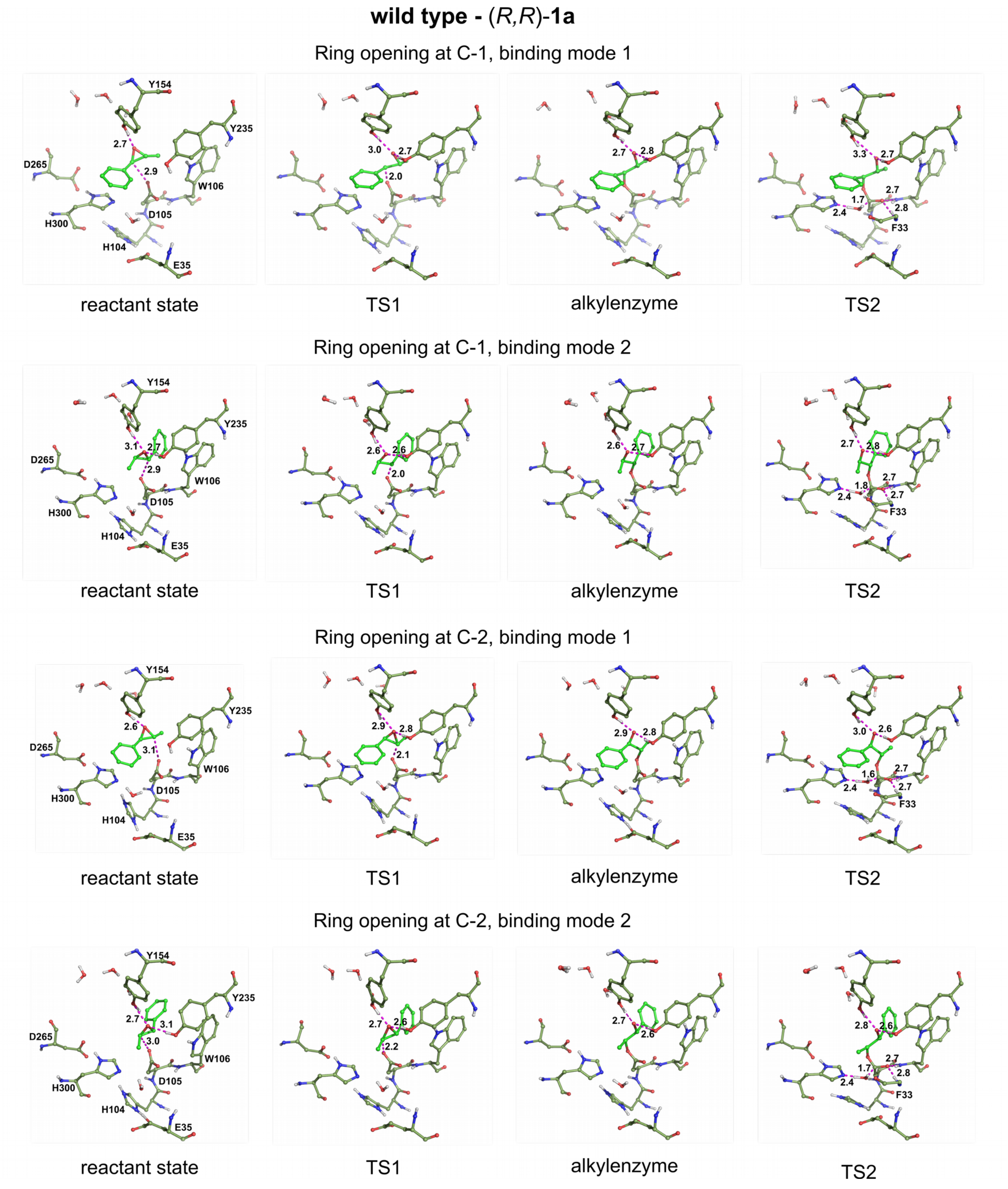

Figure S5: Key stationary points for the hydrolysis of (*R*,*R*)-1a by wild-type StEH1. Coordinates correspond to clustered structures obtained from trajectories at the respective extrema of the EVB free energy profiles.

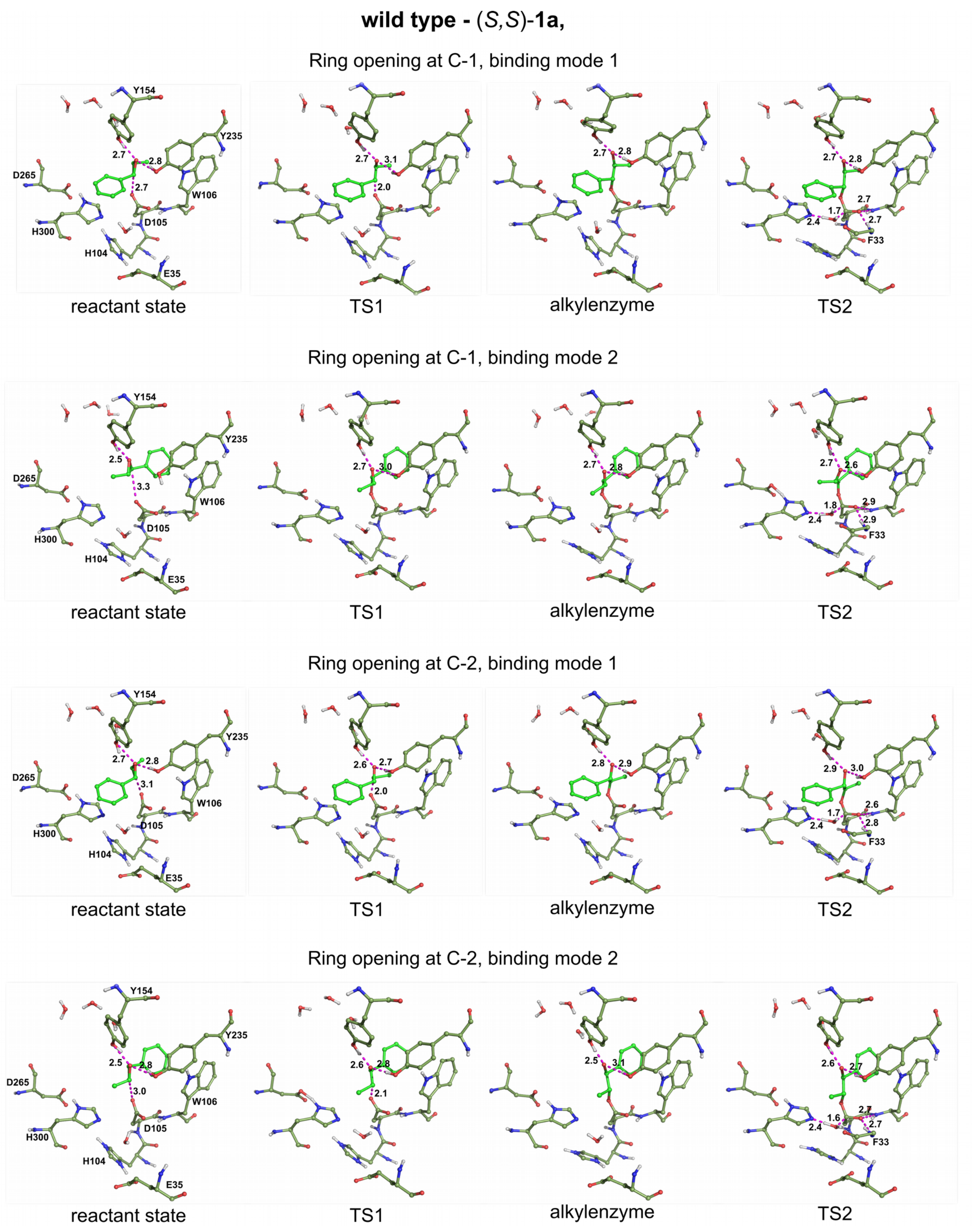

Figure S6: Key stationary points for the hydrolysis of (*S*,*S*)-**1a** by wild-type StEH1.

S4. Supplementary Tables

Table S1: A comparison of the energetics of the uncatalyzed hydrolysis of both (*R,R*)- and (*S,S*)-MeSO, from our DFT calculations and EVB calibration procedure[a]. The corresponding absolute energies for the DFT calculations are shown in **Table S2**.

		**(*R,R*)-MeSO**		**(*S,S*)-MeSO**	
		**C-1**	**C-2**	**C-1**	**C-2**
Ring Opening Step					
**DFT**	**$\Delta G^{\ddagger}$**	26.6	26.7	27.0	27.1
	**ΔG_0**	7.0	5.2	5.8	3.5
**EVB**	**$\Delta G^{\ddagger}$**	26.6 ± 0.03	26.7 ± 0.02	27.0 ± 0.02	27.1 ± 0.04
	**ΔG_0**	7.0 ± 0.03	5.2 ± 0.03	5.8 ± 0.03	3.5 ± 0.04
Hydrolysis Step					
**EVB**	**$\Delta G^{\ddagger}$**	19.0 ± 0.03	19.0 ± 0.02	19.0 ± 0.03	19.0 ± 0.02
	**ΔG_0**	10.0 ± 0.03	10.0 ± 0.02	10.0 ± 0.03	10.0 ± 0.01

[a] DFT calculations performed as described in **Section S2**. In the second step, which involves ester hydrolysis by nucleophilic attack of a water molecule on an oxirane intermediate (**Figure S2**), all EVB energetics were calibrated to a $\Delta G^{\ddagger}$ of 19 kcal mol^{-1} and a ΔG_0 of 10 kcal mol^{-1}, as described in **Section S1** and the Supporting Information of our previous work.[1] As the two binding modes described in **Figure 2** of the main text should have identical energetics in aqueous solution, we performed calculations for both SO conformations for each enantiomer, and used the conformation that gave the lowest calculated energetics (corresponding to **Mode 2** for (*R,R*)-MeSO, **Mode 1** for (S,*S*)-MeSO attack at C-1, and **Mode 2** for (S,S)-MeSO attack at C-2) for all subsequent calibration. All energies provided in kcal mol^{-1}.

Table S2: Absolute electronic energy (E_{el}, in atomic units), zero-point energy contribution ($E_{ZPE,}$ in kcal·mol^{-1}), entropies (S, in cal·mol^{-1}·K^{-1}) and frequencies (v, in cm^{-1}) for each optimized stationary point along the reaction profiles of nucleophilic attack of acetate on methylstyrene oxide. RS, TS, and PS denote reactant, transition and product states respectively.

**Species**		**E_e (a.u)**	**E_{ZPE} (kcal·mol^{-1})**	**S (cal·mol^{-1}·K^{-1})**	**v (cm^{-1})**
**(*R,R*)-MeSO**					
**RS**		-997.90450	216.004	221.289	
**(*R,R*)-C-1 Mode 1**	**TS**	-997.88316	216.199	177.559	-443.4
	**PS**	-997.91815	217.804	174.258	29.0
**(*R,R*)-C-1 Mode 2**	**TS**	-997.88296	216.123	174.539	-468.2
	**PS**	-997.91423	217.563	174.093	36.9
**(*R,R*)-C-2 Mode 1**	**TS**	-997.88481	216.307	173.799	-521.8
	**PS**	-997.92161	217.784	173.134	30.4
**(*R,R*)-C-2 Mode 2**	**TS**	-997.88363	216.231	173.862	-530.7
	**PS**	-997.92477	217.657	171.601	41.4
**(*S,S*)-MeSO**					
**(*S,S*)-C-1 Mode 1**	**TS**	-997.88356	216.414	175.694	-454.8
	**PS**	-997.91992	217.461	173.386	33.3
**(*S,S*)-C-1 Mode 2**	**TS**	-997.88257	216.654	174.971	-444.3
	**PS**	-997.91174	218.371	172.842	33.6
**(*S,S*)-C-2 Mode 1**	**TS**	-997.88434	216.528	172.575	-526.9
	**PS**	-997.92502	218.237	170.203	42.6
**(*S,S*)-C-2 Mode 2**	**TS**	-997.88432	216.309	173.468	-541.3
	**PS**	-997.92520	217.727	170.394	42.7

Table S3: Averages of key enzyme-substrate distances and substrate RMSD over our initial equilibration runs of either wild-type StEH1 or the R-C1 variant[a].

Variant	RMSD	Distances			
		Y154 – O	Y235 - O	D105 – C-1	D105 – C-2
***(R,R)*-MeSO**					
**Wild-type, Mode 1**	0.85 ± 0.14	2.98 ± 0.59	3.99 ± 0.85	3.30 ± 0.39	3.40 ± 0.29
**Wild-type, Mode 2**	1.23 ± 0.18	2.82 ± 0.34	3.05 ± 0.54	3.05 ± 0.26	3.25 ± 0.27
**R-C1, Mode 1**	0.78 ± 0.12	2.71 ± 0.15	3.84 ± 0.44	2.88 ± 0.19	3.16 ± 0.24
**R-C1, Mode 2**	1.23 ± 0.18	2.69 ± 0.20	3.38 ± 0.55	3.17 ± 0.27	3.12 ± 0.22
***(S,S)*-MeSO**					
**Wild-type, Mode 1**	0.76 ± 0.12	2.85 ± 0.44	3.06 ± 0.53	3.08 ± 0.21	3.49 ± 0.26
**Wild-type, Mode 2**	0.98 ± 0.16	2.68 ± 0.16	3.74 ± 0.88	3.43 ± 0.38	3.24 ± 0.26
**R-C1, Mode 1**	0.69 ± 0.12	2.70 ± 0.20	3.55 ± 0.52	3.00 ± 0.25	3.19 ± 0.30
**R-C1, Mode 2**	1.08 ± 0.15	2.63 ± 0.11	4.05 ± 0.71	3.35 ± 0.43	3.30 ± 0.25

[a] All values are shown in Å, and are averages and standard deviaions over 6000 data points from 30*10 ns equilibration runs on each structure. Y154-O and Y235-O denote distances between the oxygen atoms of the tyrosine side chains and the epoxide ring oxygen, and D105-C-1 and D105-C-2 indicate distances between the nucleophilic oxygen atom of the D105 side chain and the relevant carbon atom of methylstyrene oxide, as defined in **Figure 1** of the main text.

S5. Empirical Valence Bond Parameters

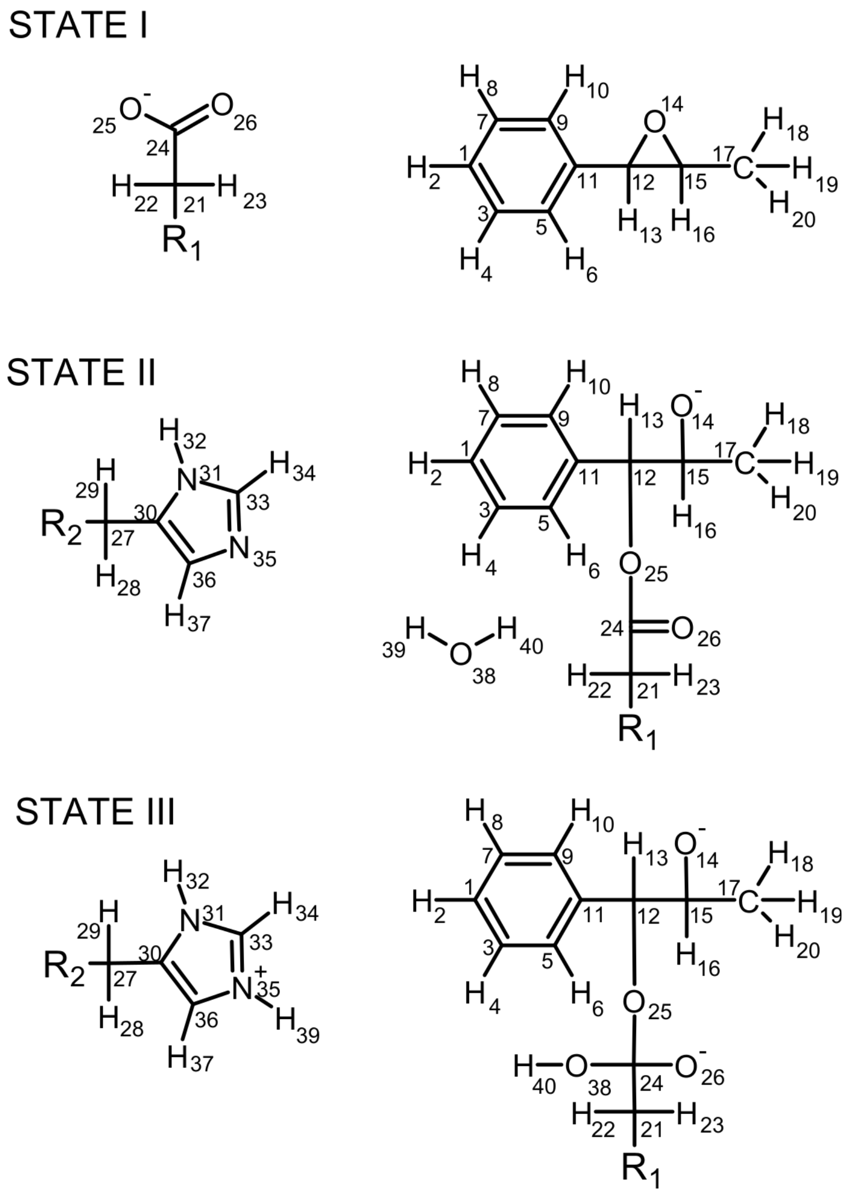

Figure S7: Atom numbering used to describe the atoms of the reacting system in the EVB parameter tables below. The different reacting states correspond to the valence bond states shown in **Figure S2**.

Table S4: EVB parameters used in this work[a].

Step I (Alkylation)				
	C-1		C-2	
	H_{ij}	α_i	H_{ij}	α_i
**(*R*,*R*)-MeSO**	57.0	-10.6	56.0	-38.1
**(S,*S*)-MeSO**	57.2	-10.0	55.1	-33.9
**Step II (Hydrolysis)**				
	C-1		C-2	
	H_{ij}	α_i	H_{ij}	α_i
**(R,*R*)-MeSO**	49.6	342.5	43.8	303.1
**(*S*,*S*)-MeSO**	46.6	317.1	42.9	301.6

[a] Both H_{ij} and α_i are constants. All values given in kcal mol^{-1}. Energy values were split over 100 sampling bins of equal width, with the first 2000 points (equal to 10 ps of simulation time) of each data collection window being skipped as equilibration, and only bins with more than 250 points being considered as statistically significant.

Table S5: Van der Waals parameters used for atoms constituting the reacting system[a].

Type	A_i ($kcal^{1/2}\cdot mol^{-1/2}\cdot Å^6$)	B_i ($kcal^{1/2}\cdot mol^{-1/2}\cdot Å^3$)	C_i ($kcal\cdot mol^{-1}$)	α_i ($Å^2$)	A_{1-4} ($kcal^{1/2}\cdot mol^{-1/2}\cdot Å^3$)	B_{1-4} ($kcal^{1/2}\cdot mol^{-1/2}\cdot Å^3$)	mass (a.u.)
**CA**	1059.13	23.67	0	0.0	748.92	16.74	12.01
**CO4**	1802.24	34.18	250	1.7	1274.38	24.17	12.01
**CT**	944.52	22.03	250	2.5	667.88	15.58	12.01
**CHMI**	944.52	22.03	250	1.7	667.88	15.58	12.01
**H**	0.00	0.00	30	2.8	0.00	0.00	1.01
**HA**	69.58	4.91	0	0.0	49.20	3.47	1.01
**HC**	49.20	3.47	0	0.0	34.79	2.45	1.01
**HC1**	109.18	6.99	0	0.0	77.20	4.94	1.01
**HC2**	84.57	5.41	0	0.0	59.80	3.83	1.01
**NA**	971.75	28.31	180	2.5	687.13	20.02	14.01
**O**	616.44	23.77	90	2.0	435.89	16.81	16.00
**OCRB**	601.15	22.26	90	1.8	425.08	15.76	16.00
**OH**	976.93	31.26	90	2.0	690.79	22.10	16.00
**OH1**	616.44	23.77	90	1.8	435.89	16.81	16.00
**OH2**	760.64	25.04	100	2.8	537.86	17.71	16.00
**OS**	445.13	18.25	90	2.0	314.75	12.91	16.00
**OS1**	445.13	18.25	0	0.0	314.75	12.91	16.00
**OT3**	762.88	24.39	100	2.8	539.44	17.25	16.00

[a] For all atoms except reacting atoms, a standard 6-12 Lennard Jones potential was used. In the case of the reacting atoms, which change bonding patterns between atoms *i* and *j*, an alternate function of the form: $V_{react} = C_i C_j\, exp\, (-\alpha_i\alpha_j r_{ij})$ was used to prevent artificial repulsion between these atoms as bonding patterns change. r_{ij} denotes the distance (Å) between atoms *i* and *j*.

Table S6: Atom types in the different VB states used to describe the reacting system, following the atom numbering shown in **Figure S7**.

		C-1 Attack		C-2 Attack	
**Atom Number**	**State I**	**State II**	**State III**	**State II**	**State III**
**1**	CA	CA	CA	CA	CA
**2**	HA	HA	HA	HA	HA
**3**	CA	CA	CA	CA	CA
**4**	HA	HA	HA	HA	HA
**5**	CA	CA	CA	CA	CA
**6**	HA	HA	HA	HA	HA
**7**	CA	CA	CA	CA	CA
**8**	HA	HA	HA	HA	HA
**9**	CA	CA	CA	CA	CA
**10**	HA	HA	HA	HA	HA
**11**	CA	CA	CA	CA	CA
**12**	CT	CT	CT	CT	CT
**13**	HC	HC	HC	HC1	HC1
**14**	OS	OH	OH	OH	OH
**15**	CT	CT	CT	CT	CT
**16**	HC	HC1	HC1	HC	HC
**17**	CT	CT	CT	CT	CT
**18**	HC	HC	HC	HC	HC
**19**	HC	HC	HC	HC	HC
**20**	HC	HC	HC	HC	HC
**21**	CT	CT	CT	CT	CT
**22**	HC	HC	HC	HC	HC
**23**	HC	HC	HC	HC	HC
**24**	CO4	CO4	CHMI	CO4	CHMI
**25**	OH1	OCRB	OS1	OCRB	OS1
**26**	OH1	OH1	OH	OH1	OH
**27**	CT	CT	CT	CT	CT
**28**	HC	HC	HC	HC	HC
**29**	HC	HC	HC	HC	HC
**30**	CA	CA	CA	CA	CA
**31**	NA	NA	NA	NA	NA
**32**	H	H	H	H	H
**33**	CA	CA	CA	CA	CA
**34**	HA	HA	HA	HA	HA
**35**	NA	NA	NA	NA	NA
**36**	CA	CA	CA	CA	CA
**37**	HA	HA	HA	HA	HA
**38**	OT3	OT3	OH2	OT3	OH2
**39**	H	H	H	H	H
**40**	H	H	H	H	H

Table S7: Partial charges of each atom used to describe the reacting system, following the atom numbering shown in **Figure S7**.

#	(*S*,*S*)-MeSO					(*R*,*R*)-MeSO				
		C-1 Attack		C-2 Attack			C-1 Attack		C-2 Attack	
	State I	State II	State III	State II	State III	State I	State II	State III	State II	State III
1	-0.162	-0.131	-0.143	-0.180	-0.181	-0.162	-0.153	-0.178	-0.177	-0.188
2	0.142	0.106	0.081	0.111	0.086	0.142	0.112	0.084	0.111	0.089
3	-0.137	-0.211	-0.238	-0.193	-0.204	-0.137	-0.208	-0.223	-0.197	-0.196
4	0.142	0.120	0.098	0.114	0.095	0.142	0.120	0.093	0.113	0.095
5	-0.137	-0.211	-0.239	-0.193	-0.204	-0.137	-0.208	-0.230	-0.197	-0.196
6	0.142	0.120	0.098	0.114	0.095	0.142	0.120	0.093	0.113	0.095
7	-0.160	-0.098	-0.065	-0.095	-0.139	-0.160	-0.051	-0.064	-0.087	-0.149
8	0.124	0.134	0.119	0.104	0.118	0.124	0.099	0.118	0.095	0.117
9	-0.160	-0.098	-0.065	-0.095	-0.139	-0.160	-0.051	-0.064	-0.087	-0.149
10	0.124	0.134	0.119	0.104	0.118	0.124	0.099	0.118	0.095	0.117
11	0.096	0.001	-0.001	-0.031	0.064	0.096	-0.021	0.002	-0.017	0.086
12	-0.036	0.190	0.228	0.434	0.388	-0.036	0.102	0.084	0.420	0.397
13	0.136	0.074	0.014	-0.106	-0.133	0.136	0.065	0.053	-0.133	-0.151
14	-0.318	-0.916	-1.006	-0.902	-0.935	-0.318	-0.921	-1.020	-0.878	-0.947
15	0.117	0.618	0.750	0.491	0.540	0.117	0.666	0.687	0.478	0.624
16	0.109	-0.146	-0.234	-0.019	-0.072	0.109	-0.097	-0.126	-0.032	-0.124
17	-0.366	-0.361	-0.363	-0.306	-0.251	-0.366	-0.529	-0.380	-0.250	-0.276
18	0.114	0.045	0.025	0.057	0.016	0.114	0.087	0.0300	0.042	0.022
19	0.114	0.045	0.025	0.056	0.016	0.114	0.087	0.0300	0.042	0.022
20	0.114	0.045	0.025	0.057	0.016	0.114	0.087	0.0300	0.042	0.022
21	-0.478	-0.326	-0.810	-0.395	-0.854	-0.480	-0.199	-0.918	-0.339	-0.851
22	0.093	0.094	0.156	0.115	0.188	0.093	0.057	0.206	0.102	0.179
23	0.093	0.119	0.208	0.127	0.225	0.093	0.068	0.248	0.111	0.202
24	0.971	0.934	1.335	1.003	1.331	0.971	0.725	1.424	0.933	1.388
25	-0.838	-0.643	-0.737	-0.715	-0.821	-0.838	-0.456	-0.691	-0.664	-0.844
26	-0.838	-0.636	-0.903	-0.657	-0.908	-0.838	-0.598	-0.940	-0.641	-0.928
27	-0.005	-0.005	-0.005	-0.005	-0.005	-0.005	-0.005	-0.005	-0.005	-0.005
28	0.060	0.060	0.060	0.060	0.060	0.060	0.060	0.060	0.060	0.060
29	0.060	0.060	0.060	0.060	0.060	0.060	0.060	0.060	0.060	0.060
30	0.015	0.015	0.215	0.015	0.215	0.015	0.015	0.215	0.015	0.215

31	-0.570	-0.570	-0.540	-0.570	-0.540	-0.570	-0.570	-0.540	-0.570	-0.540
32	0.420	0.420	0.460	0.420	0.460	0.420	0.420	0.460	0.420	0.460
33	0.295	0.295	0.385	0.295	0.385	0.295	0.295	0.385	0.295	0.385
34	0.115	0.115	0.115	0.115	0.115	0.115	0.115	0.115	0.115	0.115
35	-0.490	-0.490	-0.540	-0.490	-0.540	-0.490	-0.490	-0.540	-0.490	-0.540
36	-0.015	-0.015	0.215	-0.015	0.215	-0.015	-0.015	0.215	-0.015	0.215
37	0.115	0.115	0.115	0.115	0.115	0.115	0.115	0.115	0.115	0.115
38	-0.834	-0.834	-0.832	-0.834	-0.834	-0.834	-0.834	-0.820	-0.834	-0.855
39	0.417	0.417	0.353	0.417	0.382	0.4170	0.4170	0.3441	0.4170	0.400
40	0.417	0.417	0.460	0.417	0.460	0.417	0.417	0.460	0.417	0.460

Table S8: Bond parameters for the covalent bonds used to describe the reacting system[a].

Bond Type	E_D (kcal·mol^{-1})	α (Å^{-2})	r_0 (Å)	k_b (kcal·mol^{-1}·Å^{-2})	b (Å)
**0**	Not Set				
**1**				734	1.0800
**2**				938	1.4000
**3**				634	1.5100
**4**				680	1.0900
**5**				536	1.5290
**6**				640	1.4100
**7**				634	1.5220
**8**				428	1.3270
**9**				1140	1.2290
**10**				634	1.5040
**11**				854	1.3810
**12**				1040	1.3700
**13**				868	1.0100
**14**				954	1.3430
**15**				976	1.3350
**16**				820	1.3940
**17**				1312	1.2500
**18**				1106	0.9570
**19**				900	1.3640
**20**				1106	0.9450
**21**				560	1.5100
**22**				680	1.0880
**23**				520	1.5090
**24**				640	1.3800
**25**	80.00	2.0	1.4100		
**26**	80.00	2.0	1.3800		
**27**	284.7	1.2	1.0100		
**28**	245.8	1.5	0.9572		

[a] Morse bonds (reacting atoms): $V_{Morse} = D_e\{1-exp[-\alpha\ (r_{ij}-r_0)]\}^2$; Harmonic bonds (non-reacting atoms): $V_{Harmonic} = 0.5k\ (r_{ij}-r_0)^2$.

Table S9: Bond types in the different VB states used to describe the reacting system, following the atom numbering shown in **Figure S7**.

Atom Number		Bond Type				
			C-1 Attack		C-2 Attack	
**#1**	**#2**	**State I**	**State II**	**State III**	**State II**	**State III**
**1**	**2**	1				
**1**	**3**	2				
**1**	**7**	2				
**3**	**4**	1				
**3**	**5**	2				
**5**	**6**	1				
**5**	**11**	2				
**7**	**8**	1				
**7**	**9**	2				
**9**	**10**	1				
**9**	**11**	2				
**11**	**12**	21	3	3	3	3
**12**	**13**	22	4	4	4	4
**12**	**14**	6/25	0	0	6	6
**12**	**15**	23	5	5	5	5
**12**	**25**	0	25	6	0	0
**14**	**15**	6/25	6	6	0	0
**15**	**16**	22	4	4	4	4
**15**	**17**	21	5	5	5	5
**15**	**25**	0	0	0	25	6
**17**	**18**	4				
**17**	**19**	4				
**17**	**20**	4				
**21**	**22**	4				
**21**	**23**	4				
**21**	**24**	7	7	5	7	5
**24**	**25**	17	8	24	8	24
**24**	**26**	17	9	24	9	24
**27**	**28**	4				
**27**	**29**	4				
**27**	**30**	10				
**30**	**31**	11				
**30**	**36**	12				
**31**	**32**	13				
**31**	**33**	14				
**33**	**34**	1				
**33**	**35**	15	15	14	15	14
**35**	**25**	0	0	27	0	27
**35**	**36**	16	16	11	16	11
**36**	**37**	1				
**38**	**24**	0	0	26	0	26
**38**	**39**	18	28	0	28	0
**38**	**40**	18	18	20	18	20
**R1**	**21**	5				

**R2**	**27**	5

Table S10: Angle parameters used for bending adjacent bonds i the reacting system[a].

Angle Type	k_a (kcal·mol^{-1}·rad^{-2})	Θ_0 (°)	Angle Type	k_a (kcal·mol^{-1}·rad^{-2})	Θ_0 (°)
**0**	No Set				
**1**	70.0	120.00	**18**	140.0	110.00
**2**	126.0	120.00	**19**	140.0	111.00
**3**	140.0	120.00	**20**	70.0	128.20
**4**	70.0	109.50	**21**	116.7	112.70
**5**	126.0	114.00	**22**	140.0	117.00
**6**	100.0	109.50	**23**	160.0	126.00
**7**	75.0	110.70	**24**	200.0	104.52
**8**	66.0	107.80	**25**	70.0	113.00
**9**	126.0	111.10	**26**	75.0	117.20
**10**	162.0	111.40	**27**	75.0	117.20
**11**	160.0	120.40	**28**	60.0	60.00
**12**	166.0	123.40	**29**	185.2	111.55
**13**	166.0	116.90	**30**	120.0	109.50
**14**	140.0	121.60	**31**	70.0	130.70
**15**	140.0	130.70	**32**	110.0	108.50
**16**	140.0	106.30	**33**	66.0	107.8
**17**	140.0	109.80	**34**	70	114.3

[a] Angle potential: $V_{angle}=0.5\sum k\ (\Theta - \Theta_0)^2$

Table S11: Angle types of the different VB states used to describe the reacting system following the atom numbering shown in **Figure S7**.

Atom Number			Angle Type				
				C-1 Attack		C-2 Attack	
**#1**	**#2**	**#3**	**State I**	**State II**	**State III**	**State II**	**State III**
**1**	**3**	**4**	1				
**1**	**3**	**5**	2				
**1**	**7**	**8**	1				
**1**	**7**	**9**	2				
**2**	**1**	**3**	1				
**2**	**1**	**7**	1				
**3**	**1**	**7**	2				
**3**	**5**	**6**	1				
**3**	**5**	**11**	2				
**4**	**3**	**5**	1				
**5**	**11**	**9**	2				
**5**	**11**	**12**	3				
**6**	**5**	**11**	1				
**7**	**9**	**10**	1				
**7**	**9**	**11**	2				
**8**	**7**	**9**	1				
**9**	**11**	**12**	3				
**10**	**9**	**11**	1				
**11**	**12**	**13**	4				
**11**	**12**	**14**	6	0	0	6	6
**11**	**12**	**15**	5				
**11**	**12**	**25**	0	6	6	0	0
**12**	**14**	**15**	28	0	0	0	0
**12**	**15**	**14**	28	6	6	0	0
**12**	**15**	**16**	27	7	7	7	7
**12**	**15**	**17**	27	21	21	21	21
**15**	**17**	**18**	7				
**15**	**17**	**19**	7				
**15**	**17**	**20**	7				
**14**	**15**	**17**	27	6	0	6	0

**12**	**15**	**25**	0	0	0	6	6
**13**	**12**	**14**	26	0	0	4	4
**12**	**25**	**24**	0	13	30	0	0
**13**	**12**	**15**	27	7	7	7	7
**13**	**12**	**25**	0	4	4	0	0
**14**	**12**	**15**	28	0	0	6	6
**14**	**15**	**16**	26	4	4	0	0
**15**	**12**	**25**	0	6	6	0	0
**15**	**25**	**24**	0	0	0	13	30
**16**	**15**	**17**	34	7	7	7	7
**16**	**15**	**25**	0	0	0	4	4
**17**	**15**	**25**	0	0	0	6	6
**21**	**24**	**25**	22	10	6	10	6
**21**	**24**	**26**	22	11	6	11	6
**21**	**24**	**38**	0	0	6	0	6
**22**	**21**	**23**	8				
**22**	**21**	**24**	4	4	7	4	7
**23**	**21**	**24**	4	4	7	4	7
**24**	**38**	**40**	0	0	32	0	32
**25**	**24**	**26**	23	12	29	12	29
**25**	**24**	**38**	0	0	29	0	29
**26**	**24**	**38**	0	0	29	0	29
**39**	**38**	**40**	24	24	0	24	0
**27**	**30**	**31**	14				
**27**	**30**	**36**	15				
**28**	**27**	**29**	8				
**28**	**27**	**30**	4				
**29**	**27**	**30**	4				
**30**	**31**	**32**	1				
**30**	**31**	**33**	17				
**30**	**36**	**35**	19	19	16	19	16
**30**	**36**	**37**	20	20	31	20	31
**31**	**30**	**36**	16				
**31**	**33**	**34**	1				
**31**	**33**	**35**	3				
**32**	**31**	**33**	1				

**33**	**35**	**39**	0	0	1	0	1
**33**	**35**	**36**	18	18	17	18	17
**34**	**33**	**35**	1				
**35**	**36**	**37**	1				
**36**	**35**	**39**	0	0	1	0	1
**R1**	**21**	**22**	7				
**R1**	**21**	**23**	7				
**R1**	**21**	**24**	9	9	21	9	21
**R2**	**27**	**28**	7				
**R2**	**27**	**29**	7				
**R2**	**27**	**30**	5				

Table S12: Torsion parameters used to describe the reacting system[a].

Torsion Type	V_1	V_2	V_3	Torsion Type	V_1	V_2	V_1
	0.5·barrier height ($kcal \cdot mol^{-1}$)				0.5·barrier height ($kcal \cdot mol^{-1}$)		
**0**	Not Set						
**1**	0.0000	3.6250	0.0000	**16**	0.0000	0.0000	0.2100
**2**	0.8550	-0.2500	0.3310	**17**	0.0000	1.4000	0.0000
**3**	0.0000	0.0000	0.2310	**18**	0.0000	5.3750	0.0000
**4**	-0.8485	-0.2280	0.2925	**19**	0.0000	2.3250	0.0000
**5**	0.0000	0.0000	0.2340	**20**	0.0000	5.0000	0.0000
**6**	0.0000	0.0000	0.1500	**21**	0.0000	2.4000	0.0000
**7**	2.1590	0.0000	0.0000	**22**	0.0000	0.4100	0.0000
**8**	-0.6100	-0.0630	0.2110	**23**	0.3250	-0.1250	0.3350
**9**	0.0000	0.0000	0.0990	**24**	0.0000	0.0000	0.3800
**10**	0.0000	0.0000	-0.2760	**25**	-0.6680	0.0000	0.0000
**11**	-0.1390	0.6140	-0.3470	**26**	-0.2610	-1.0090	0.9980
**12**	0.0000	0.0000	0.0660	**27**	-0.1780	-0.0870	0.2460
**13**	2.3350	2.5620	0.0000	**28**	-0.6280	-0.9030	0.0020
**14**	0.0000	2.5620	0.0000	**29**	0.0000	1.6000	0.0000
**15**	1.1830	-0.1310	0.2530	**30**	0.650	-0.025	0.100

[a] Torsion angle potential: $V_{torsion} = V_1\ (1+\cos(n\varphi\ -\delta)) + V_2\ (1+\cos 2(n\varphi\ -\delta)) + V_3\ (1+\cos 3(n\varphi\ -\delta))$, *n* is the periodicity (number of maxima per turn) and δ is the phase shift.

Table S13: Torsion types in the different VB states used to describe the reacting system, following the atom numbering shown in **Figure S7**.

Atom Number				Torsion Type				
					C-1 Attack		C-2 Attack	
#1	#2	#3	#4	State I	State II	State III	State II	State III
1	3	5	6	1				
1	3	5	11	1				
1	7	9	10	1				
1	7	9	11	1				
2	1	3	4	1				
2	1	3	5	1				
2	1	7	8	1				
2	1	7	9	1				
3	1	7	8	1				
3	1	7	9	1				
3	5	11	9	1				
3	5	11	12	1				
4	3	5	6	1				
4	3	5	11	1				
6	5	11	9	1				
6	5	11	12	1				
7	1	3	4	1				
7	1	3	5	1				
7	9	11	5	1				
7	9	11	12	1				
8	7	9	10	1				
8	7	9	11	1				
10	9	11	5	1				
10	9	11	12	1				
11	12	14	15	23	0	0	0	0
11	12	15	14	2	2	2	0	0
11	12	15	16	3				
11	12	15	17	30				
12	15	17	18	6				
12	15	17	19	6				
12	15	17	20	6				
13	15	17	18	6				
13	15	17	19	6				
13	15	17	20	6				
14	15	17	18	5	5	5	0	0
14	15	17	19	5	5	5	0	0
14	15	17	20	5	5	5	0	0
16	15	17	18	6				
16	15	17	19	6				
16	15	17	20	6				
13	12	15	25	0	0	0	5	5
13	12	25	24	0	9	24	0	0
14	12	15	16	5	0	0	5	5
14	12	15	17	2	0	0	2	2
14	12	15	25	0	0	0	7	7

**15**	**12**	**25**	**24**	0	8	23	0	0
**16**	**15**	**25**	**24**	0	0	0	9	24
**17**	**15**	**25**	**24**	0	0	0	2	8
**21**	**24**	**25**	**12**	0	13	23	0	0
**21**	**24**	**25**	**15**	0	0	0	13	23
**21**	**24**	**25**	**38**	0	0	27	0	27
**22**	**21**	**24**	**25**	0	12	5	12	5
**22**	**21**	**24**	**26**	0	0	5	0	5
**22**	**21**	**24**	**38**	0	0	5	0	5
**23**	**21**	**24**	**25**	0	12	5	12	5
**23**	**21**	**24**	**26**	0	0	5	0	5
**23**	**21**	**24**	**38**	0	0	5	0	5
**25**	**12**	**15**	**14**	0	7	7	0	0
**25**	**12**	**15**	**16**	0	5	5	0	0
**25**	**12**	**15**	**17**	0	2	2	0	0
**25**	**15**	**17**	**18**	0	0	0	5	5
**25**	**15**	**17**	**19**	0	0	0	5	5
**25**	**15**	**17**	**20**	0	0	0	5	5
**25**	**24**	**38**	**40**	0	0	28	0	28
**26**	**24**	**25**	**12**	0	14	16	0	0
**26**	**24**	**25**	**15**	0	0	0	14	26
**26**	**24**	**38**	**40**	0	0	28	0	28
**38**	**24**	**25**	**12**	0	0	26	0	0
**38**	**24**	**25**	**15**	0	0	0	0	26
**39**	**35**	**36**	**30**	0	0	17	0	17
**39**	**35**	**36**	**37**	0	0	29	0	29
**27**	**30**	**31**	**32**	17				
**27**	**30**	**31**	**33**	17				
**27**	**30**	**36**	**35**	18				
**27**	**30**	**36**	**37**	18				
**28**	**27**	**30**	**31**	16				
**29**	**27**	**30**	**31**	16				
**30**	**31**	**33**	**34**	19				
**30**	**31**	**33**	**35**	19				
**31**	**30**	**36**	**35**	18				
**31**	**30**	**36**	**37**	18				
**31**	**33**	**35**	**36**	20	20	19	20	19
**31**	**33**	**35**	**39**	0	0	19	0	19
**32**	**31**	**33**	**34**	19				
**32**	**31**	**33**	**35**	19				
**33**	**35**	**36**	**30**	21	21	17	21	17
**33**	**35**	**36**	**37**	21	21	29	21	29
**34**	**33**	**35**	**36**	20	20	19	20	19
**34**	**33**	**35**	**39**	0	0	19	0	19
**36**	**30**	**31**	**32**	17				
**36**	**30**	**31**	**33**	17				
**R1**	**21**	**24**	**25**	22	10	25	20	25
**R1**	**21**	**24**	**26**	22	11	25	11	25
**R1**	**21**	**24**	**38**	0	0	25	0	25
**R2**	**27**	**30**	**31**	15				

Table S14: Improper torsion parameters used to describe the reacting system[a].

Improper Type	k_a (kcal·mol^{-1} rad^{-2})	τ_0 (°)
0	Not Set	
1	1.1	180
2	10.5	180
3	1.0	180

[a] Improper torsion potential: $V_{torsion}=k\ (\tau\ -\tau_0)^2$.. k_i is the force constant and τ is the equilibrium angle (in degrees).

Table S15: Improper torsion angles of the VB states used to describe the reacting system, following the atom numbering shown in **Figure S7**.

Atom Number				Improper Type		
**#1**	**#2**	**#3**	**#4**	**State I**	**State II**	**State III**
**1**	**3**	**5**	**4**		1	
**1**	**7**	**9**	**8**		1	
**3**	**1**	**7**	**2**		1	
**11**	**5**	**3**	**6**		1	
**11**	**9**	**7**	**10**		1	
**12**	**11**	**5**	**9**		1	
**21**	**24**	**25**	**26**	2	2	0
**27**	**30**	**36**	**31**		1	
**30**	**31**	**33**	**32**		3	
**30**	**36**	**37**	**35**		1	
**33**	**35**	**36**	**39**	0	0	3
**34**	**33**	**31**	**35**		1	

S6. Cartesian Coordinates for Key Stationary Points

Optimized stationary points along the reaction profiles of the nucleophilic attack of propionate on (S,S)-MeSO and (R,R)-MeSO through all previously reaction pathways studied in this work. RS, TS, and PS denote reactant, transition and product states respectively. The reactant state molecules are only shown once at infinite separation. Each of the individual reacting species were microsolvated with two water molecules, and each of the reacting complexes were microsolvated with four water molecules.

Reactant

Methylstyrene oxide

C	1.463926	-0.472829	1.184325
C	0.603106	-0.771946	0.033357
O	1.500931	0.367770	0.007762
C	2.704629	-1.260800	1.481139
H	1.002754	0.029378	2.033363
H	0.935546	-1.570921	-0.627950
C	-0.859440	-0.494217	-0.012119
C	-1.438821	0.568041	0.691548
H	-0.819529	1.231421	1.289327
H	-3.250428	1.617811	1.172518
C	-2.811912	0.787740	0.625937
C	-3.623368	-0.054773	-0.136383
H	-4.694728	0.118201	-0.184548
C	-3.052828	-1.115939	-0.836753
H	-3.676378	-1.774491	-1.434629
C	-1.676040	-1.330444	-0.778136
H	-1.231189	-2.153643	-1.331896
O	0.738299	3.076795	-0.263134
H	0.941977	2.129916	-0.129727
H	-0.227422	3.119775	-0.226976
O	3.662958	-0.030119	-1.756348
H	3.987534	-0.914623	-1.538651
H	2.912227	0.108043	-1.143788
H	2.479402	-2.020838	2.236881
H	3.485782	-0.604767	1.879222
H	3.081433	-1.759825	0.583125

Propionate

C	-0.893232	1.791987	0.046480
H	-1.500421	1.579853	-0.839543
H	-1.503566	1.589003	0.932548
H	-0.654422	2.861331	0.041121
C	0.389416	0.972296	0.052395
H	1.006459	1.234950	0.920886
H	0.999024	1.211051	-0.827874
C	0.216952	-0.543712	0.077985
O	1.280214	-1.239159	0.059649
O	-0.938735	-1.052983	0.120845
O	-3.602847	-0.369476	-0.055455
H	-3.835218	-0.648253	-0.951434
H	-2.628422	-0.498467	-0.000905
O	3.700715	0.011716	-0.139971
H	3.512907	0.960208	-0.150718
H	2.810068	-0.413885	-0.065794

(R,R)-MeSO C-1 Mode 1

TS

O	1.115116	-0.692438	0.372823
C	-0.479512	0.611004	0.000297
C	-0.677100	1.312650	1.276210
O	-1.559886	2.143644	0.554235
H	-1.201911	0.726019	2.040175
C	0.524061	2.011216	1.876981
H	0.140109	1.137183	-0.714899
C	-1.390423	-0.406245	-0.538730
C	-2.217834	-1.181728	0.283729
H	-2.215392	-1.034612	1.359219
C	-3.056558	-2.141098	-0.274370
H	-3.698416	-2.735634	0.368980
C	-3.071658	-2.340654	-1.656108
H	-3.727831	-3.090580	-2.088568
H	-2.251940	-1.733050	-3.555517
C	-2.243869	-1.578650	-2.480637
C	-1.406268	-0.617046	-1.922977
H	-0.757794	-0.021034	-2.560234
C	2.249715	-0.380689	-0.118767
O	2.437673	0.622919	-0.855277
C	3.440690	-1.281849	0.175696
H	3.768060	-1.680894	-0.792978
H	4.253419	-0.632462	0.521970
C	3.201144	-2.414028	1.164784
H	2.944320	-2.028943	2.156628
H	2.392043	-3.073047	0.834008

H	4.109133	-3.018393	1.264781
O	-0.273863	3.945730	-1.018500
H	0.571888	3.515086	-1.205725
H	-0.742092	3.304107	-0.422614
O	-4.137074	1.467165	0.917653
H	-4.210867	0.544302	0.636355
H	-3.177324	1.691013	0.777413
O	0.288772	-1.777605	2.806191
H	0.619595	-1.419796	1.954592
H	-0.518711	-1.274772	2.979121
O	4.940414	0.989802	-1.933101
H	4.046900	0.850392	-1.538106
H	5.468311	0.256894	-1.587156
H	1.183424	1.289125	2.370589
H	0.185130	2.736699	2.624936
H	1.096392	2.541353	1.108206

PS

O	1.041896	-0.547758	0.126677
C	-0.079254	0.386561	0.062741
C	-0.468595	0.833642	1.492969
O	-1.491482	1.777133	1.407295
H	-0.823580	-0.067919	2.026623
C	0.751637	1.378028	2.239232
H	0.248855	1.265493	-0.493811
C	-1.202490	-0.296160	-0.672795
C	-1.602286	-1.594321	-0.338602
H	-1.074723	-2.141652	0.437641
C	-2.677755	-2.191923	-0.991899
H	-2.978064	-3.201159	-0.724143
C	-3.373567	-1.493984	-1.981082
H	-4.215927	-1.958435	-2.486014
H	-3.513225	0.349067	-3.090143
C	-2.979420	-0.200329	-2.319735
C	-1.895647	0.391751	-1.670812
H	-1.589882	1.400838	-1.936345
C	2.216888	-0.173645	-0.366121
O	2.425586	0.947153	-0.820701
C	3.272086	-1.247149	-0.369106
H	3.406656	-1.509646	-1.426681
H	4.204311	-0.765241	-0.057026
C	3.000065	-2.494044	0.459758
H	2.941661	-2.256202	1.526109
H	2.072436	-2.988912	0.157657
H	3.821352	-3.203921	0.321216
O	-0.799683	3.841016	-0.074095
H	0.076913	3.625180	-0.420615
H	-1.049362	3.037528	0.487314

O	-3.840390	0.626214	1.316256
H	-3.836391	0.034662	0.550659
H	-2.921068	1.053094	1.313984
O	0.476324	-2.412640	2.654352
H	0.726725	-1.863093	1.894131
H	-0.451864	-2.187154	2.807958
O	4.996758	1.415148	-1.886078
H	4.100930	1.265159	-1.520562
H	5.456495	0.578241	-1.731357
H	1.539581	0.625226	2.359378
H	0.444527	1.706127	3.238635
H	1.171599	2.242033	1.708462

(R,R)-MeSO C-1 Mode 2

TS

C	1.382503	2.294911	2.088097
H	2.040868	2.970955	2.626063
C	1.295013	2.367887	0.696694
H	1.883239	3.099531	0.150057
C	0.623983	1.356279	2.787771
H	0.686754	1.300480	3.870394
C	0.455138	1.502890	0.003657
H	0.396576	1.567629	-1.077897
C	-0.212246	0.486282	2.095027
H	-0.799796	-0.252127	2.634274
C	-0.302944	0.549609	0.698577
C	-1.133341	-0.440779	0.001715
H	-1.671314	-1.141682	0.632000
O	-2.775721	0.542899	-0.675118
C	-1.767781	-0.202376	-1.313825
H	-1.160175	0.418521	-1.984571
C	-2.264668	-1.430161	-2.051399
O	0.272142	-1.880347	-0.215084
C	1.390634	-1.443516	-0.695320
O	1.486882	-0.961860	-1.841121
C	2.581176	-1.524679	0.238502
H	2.267224	-1.049374	1.177522
H	2.733009	-2.584870	0.478307
C	3.863478	-0.891420	-0.280106
H	4.670577	-1.032714	0.446468
H	3.735416	0.184903	-0.435804
H	4.178916	-1.338069	-1.228664
H	-2.460655	2.193593	-0.678352
O	-2.329430	3.182544	-0.688900
H	-1.432951	3.309471	-0.348188
H	-3.937329	-0.412935	0.124282
O	-4.616814	-0.949141	0.616122

H	-4.186886	-1.803765	0.756775
H	0.229726	-2.595026	1.406115
O	0.150451	-3.027152	2.287825
H	0.383643	-2.330703	2.916724
H	2.343981	0.554087	-2.374487
O	2.702537	1.392689	-2.733958
H	2.697955	1.993906	-1.976259
H	-1.434926	-1.968805	-2.520374
H	-2.961534	-1.117614	-2.837143
H	-2.788365	-2.112002	-1.371693

PS

C	-0.455126	3.646003	0.540619
H	-0.434010	4.722667	0.683322
C	-0.655134	3.110742	-0.731920
H	-0.788661	3.769661	-1.585150
C	-0.286128	2.789637	1.629395
H	-0.135192	3.195607	2.625640
C	-0.678152	1.728571	-0.918201
H	-0.833429	1.332743	-1.916584
C	-0.322135	1.410391	1.440078
H	-0.203215	0.745070	2.292562
C	-0.513208	0.864282	0.166211
C	-0.537368	-0.647500	0.037001
H	-1.141309	-1.053031	0.854105
O	-2.497048	-1.013162	-1.264817
C	-1.116842	-1.241385	-1.263561
H	-0.643118	-0.747207	-2.127067
C	-0.785688	-2.734106	-1.364094
O	0.785356	-1.182776	0.391754
C	1.869508	-0.882840	-0.319522
O	1.834175	-0.236764	-1.356869
C	3.120335	-1.417778	0.320864
H	3.219897	-0.910214	1.288644
H	2.950935	-2.476147	0.548811
C	4.366070	-1.222890	-0.530638
H	5.236345	-1.633088	-0.009541
H	4.554232	-0.162101	-0.723328
H	4.272647	-1.735638	-1.493266
H	-3.246062	0.310219	-0.604126
O	-3.830426	1.036329	-0.228216
H	-3.211928	1.731915	0.034778
H	-3.183246	-1.923428	-0.097727
O	-3.633869	-2.471065	0.623404
H	-2.901668	-2.899841	1.087053
H	0.830395	-1.716235	2.222544
O	0.706062	-1.934879	3.163407
H	0.006083	-1.332233	3.451155

H	2.536050	1.567833	-0.985695
O	2.793009	2.462013	-0.700621
H	1.959144	2.860602	-0.408652
H	0.285474	-2.914609	-1.511681
H	-1.318266	-3.155517	-2.223888
H	-1.105962	-3.273365	-0.463641

(R,R)-MeSO C-2 Mode 1

TS

O	0.796441	-1.539595	-1.220605
C	-1.230060	-1.229255	-0.943410
C	-1.684812	-2.650479	-0.891676
H	-1.175048	-0.744050	-1.909891
C	-1.356629	-0.334167	0.215766
H	-1.301510	-0.865318	1.174073
O	-2.681853	-0.174503	-0.238364
C	-0.513811	0.915014	0.269929
C	-0.425059	1.781895	-0.824622
H	-0.975173	1.563142	-1.736797
C	0.358701	2.930560	-0.754100
H	0.422706	3.593527	-1.612697
C	1.060535	3.230258	0.416237
H	1.673340	4.125827	0.469487
C	0.968812	2.377001	1.513603
H	1.510251	2.601934	2.428603
C	0.183871	1.225854	1.437564
H	0.115394	0.559245	2.294349
O	1.234488	-2.077880	0.903234
C	1.597191	-1.690802	-0.234875
C	3.052123	-1.335885	-0.487681
H	3.306759	-1.595958	-1.519657
H	3.685324	-1.922124	0.184809
C	3.292494	0.161907	-0.258138
H	2.630588	0.770862	-0.883498
H	3.118619	0.432842	0.787546
H	4.327238	0.417984	-0.507805
O	1.359520	0.171793	-3.321509
H	1.202529	-0.443520	-2.569770
H	1.326716	1.049098	-2.914512
O	2.508045	-1.015025	3.125244
H	2.098455	-1.401627	2.318052
H	2.410411	-0.060065	3.004628
O	-4.219678	-1.724677	1.336057
H	-3.733081	-2.559110	1.379861
H	-3.664502	-1.149734	0.743984
O	-3.649928	2.357612	0.031067
H	-2.876458	2.934144	-0.044527

H	-3.280311	1.445816	-0.078507
H	-1.109739	-3.246829	-1.603202
H	-2.736015	-2.710264	-1.189068
H	-1.572914	-3.069028	0.111967

PS

O	-0.658598	-1.466409	0.963953
C	0.803926	-1.361189	1.036381
C	1.448378	-2.723970	0.863878
H	0.935832	-1.020620	2.066665
C	1.386088	-0.263586	0.113401
H	1.442287	-0.675976	-0.908143
O	2.663856	0.060367	0.579355
C	0.468522	0.951969	0.051328
C	0.162462	1.681914	1.205852
H	0.568328	1.367353	2.164817
C	-0.660090	2.803679	1.141701
H	-0.895870	3.353746	2.049092
C	-1.180806	3.222790	-0.085667
H	-1.821605	4.098886	-0.135950
C	-0.870905	2.511630	-1.242880
H	-1.267780	2.830152	-2.203382
C	-0.052446	1.382790	-1.169625
H	0.185260	0.828897	-2.075474
O	-0.744276	-1.964569	-1.228645
C	-1.305579	-1.710422	-0.169710
C	-2.796021	-1.591146	-0.010535
H	-3.084974	-2.000287	0.961700
H	-3.267555	-2.183980	-0.797820
C	-3.235877	-0.123450	-0.112957
H	-2.744630	0.493651	0.646136
H	-3.002226	0.289283	-1.098752
H	-4.317264	-0.057260	0.038971
O	-1.885095	-0.206095	3.265166
H	-1.509061	-0.646841	2.482130
H	-1.842273	0.734797	3.042878
O	-1.914808	-0.738040	-3.524613
H	-1.550723	-1.204305	-2.747054
H	-1.899149	0.193990	-3.265085
O	4.286956	-1.428355	-0.848721
H	3.815680	-2.267223	-0.943495
H	3.671385	-0.858032	-0.284371
O	3.384726	2.359024	-0.511991
H	2.548686	2.820076	-0.668829
H	3.106662	1.480305	-0.102456
H	0.965546	-3.460356	1.514187
H	2.501288	-2.657642	1.152721
H	1.394158	-3.072059	-0.171525

(R,R)-MeSO C-2 Mode 2

TS

C	1.780182	2.670813	1.452186
H	2.622015	3.314570	1.692868
C	1.610633	1.457495	2.121296
H	2.321759	1.150525	2.883212
C	0.861797	3.052729	0.473561
H	0.989277	3.994196	-0.053587
C	0.523868	0.640346	1.817957
H	0.391688	-0.301707	2.345200
C	-0.218929	2.227049	0.165245
H	-0.928610	2.529411	-0.598643
C	-0.398088	1.016387	0.836724
C	-1.550677	0.099227	0.507892
H	-1.825890	-0.494958	1.390000
O	-2.662680	0.683661	-0.120804
C	-1.475755	-0.704838	-0.719862
H	-1.052859	-0.202362	-1.582201
C	-2.348786	-1.894860	-0.945627
O	0.248405	-1.803196	-0.411242
C	1.374922	-1.292543	-0.734783
O	1.504502	-0.410484	-1.619944
C	2.602864	-1.789940	0.009785
H	2.939058	-0.964120	0.650798
H	2.315103	-2.615846	0.665666
C	3.736306	-2.212028	-0.925295
H	4.596923	-2.555779	-0.342307
H	4.065179	-1.379644	-1.554774
H	3.420705	-3.032458	-1.579567
H	-4.072849	-0.023964	0.506584
O	-4.889017	-0.437978	0.895306
H	-4.810660	-1.375354	0.671381
H	-2.667242	1.541062	-1.641804
O	-2.744441	2.008627	-2.509024
H	-1.834398	2.057721	-2.832236
H	-0.082858	-2.588329	1.149154
O	-0.333356	-3.026120	1.994256
H	-0.825804	-2.346968	2.475704
H	2.842994	0.740755	-1.366864
O	3.519675	1.436056	-1.197894
H	3.175088	1.907075	-0.424585
H	-1.839700	-2.604744	-1.600888
H	-3.268971	-1.585568	-1.449685
H	-2.604683	-2.387065	-0.003127

PS

C	1.939754	2.719066	1.292310
H	2.800623	3.343461	1.516411
C	1.695020	1.565149	2.037024
H	2.366291	1.284531	2.844298
C	1.069234	3.066483	0.256342
H	1.256661	3.960116	-0.333064
C	0.581346	0.774397	1.752999
H	0.391196	-0.119430	2.343609
C	-0.036704	2.267267	-0.027059
H	-0.709629	2.544499	-0.834300
C	-0.298896	1.115593	0.721754
C	-1.508203	0.239667	0.401212
H	-1.711869	-0.370779	1.302812
O	-2.629849	0.974699	0.038150
C	-1.144532	-0.771796	-0.715618
H	-0.832689	-0.235506	-1.614596
C	-2.252107	-1.755609	-1.028391
O	-0.015114	-1.568793	-0.246871
C	1.212998	-1.354777	-0.710935
O	1.459757	-0.574301	-1.621692
C	2.244469	-2.163767	0.025427
H	2.512455	-1.570727	0.910872
H	1.771814	-3.078371	0.394078
C	3.481789	-2.474967	-0.808048
H	4.182771	-3.069360	-0.214420
H	3.995193	-1.562813	-1.124142
H	3.218834	-3.050430	-1.701733
H	-3.929079	0.185047	0.534619
O	-4.752451	-0.288627	0.887162
H	-4.661495	-1.199167	0.576014
H	-2.703336	1.366989	-1.536333
O	-2.789706	1.636133	-2.505595
H	-1.881122	1.625529	-2.836037
H	-0.357237	-2.416035	1.461743
O	-0.572551	-2.798065	2.331266
H	-0.967218	-2.059231	2.815742
H	2.960102	0.542544	-1.380673
O	3.643084	1.214127	-1.191973
H	3.240661	1.758893	-0.498172
H	-1.915563	-2.480131	-1.776881
H	-3.119013	-1.225251	-1.430390
H	-2.558710	-2.296009	-0.125135

(S,S)-MeSO C-1 Mode 1

TS

C	-3.589796	-2.063916	0.907810
H	-4.382655	-2.755310	1.178325
C	-2.727053	-1.572584	1.887780
H	-2.843915	-1.880159	2.922673
C	-3.432059	-1.669497	-0.422437
H	-4.098508	-2.056155	-1.187726
C	-1.713372	-0.685205	1.537115
H	-1.037046	-0.301071	2.296516
C	-2.417495	-0.784307	-0.773391
H	-2.300931	-0.490094	-1.811660
C	-1.555128	-0.277805	0.207470
C	-0.463133	0.651718	-0.115784
H	0.189807	0.942750	0.697767
O	-1.278244	2.391853	-0.423581
C	-0.483650	1.576207	-1.257748
H	-1.038983	1.206345	-2.128048
C	0.827747	2.194921	-1.692227
O	0.894217	-0.843873	-0.671511
C	2.117964	-0.713368	-0.310470
O	2.521095	0.189424	0.457793
C	3.074938	-1.747026	-0.885997
C	4.529193	-1.601006	-0.460323
H	2.687709	-2.733781	-0.604062
H	2.984326	-1.690346	-1.977526
H	5.133625	-2.389278	-0.921718
H	4.638134	-1.685441	0.625965
H	4.941268	-0.635423	-0.771278
H	-2.960368	2.192023	-0.562363
O	-3.951511	2.124206	-0.614674
H	-4.135722	1.196552	-0.408922
H	-0.369220	3.260663	0.756302
O	0.142490	3.733103	1.463756
H	0.954863	3.215189	1.548539
H	0.207767	-2.516783	-0.462773
O	-0.146952	-3.420591	-0.316950
H	-1.024784	-3.271983	0.062908
H	4.081260	0.704727	1.146526
O	4.857700	1.106907	1.595814
H	5.594979	0.523723	1.369889
H	0.622419	3.059805	-2.332963
H	1.425891	1.477099	-2.264033
H	1.410800	2.529916	-0.828305

PS

C	-3.456660	-2.146211	0.515325
H	-4.298757	-2.807240	0.699992
C	-2.932526	-1.373229	1.550534
H	-3.363649	-1.429566	2.546130
C	-2.892523	-2.069052	-0.759936
H	-3.295483	-2.669413	-1.570806
C	-1.847441	-0.529298	1.311299
H	-1.435176	0.065138	2.122685
C	-1.814241	-1.219906	-0.996798
H	-1.383701	-1.161262	-1.993104
C	-1.282171	-0.443284	0.038262
C	-0.128920	0.491991	-0.216802
H	0.289032	0.837716	0.730948
O	-1.522015	2.439807	-0.434384
C	-0.515312	1.720121	-1.076126
H	-0.876381	1.321464	-2.044707
C	0.710722	2.594497	-1.351371
O	0.893620	-0.268965	-0.920359
C	2.143407	-0.285441	-0.463916
O	2.498988	0.314661	0.541626
C	3.034222	-1.127992	-1.335237
C	4.482249	-1.177364	-0.871510
H	2.594757	-2.132104	-1.368059
H	2.958013	-0.729317	-2.353656
H	5.063778	-1.806300	-1.552316
H	4.564047	-1.600557	0.134568
H	4.931874	-0.179459	-0.863972
H	-2.984112	1.830860	-0.678049
O	-3.936290	1.526488	-0.849219
H	-3.950787	0.599066	-0.573995
H	-1.157381	2.784595	1.103891
O	-0.963727	3.046922	2.061768
H	-0.587611	2.249873	2.459190
H	0.539795	-2.684158	0.171268
O	0.285983	-3.400941	0.770504
H	-0.625072	-3.180987	1.015793
H	4.118288	0.514550	1.425428
O	4.898009	0.699862	1.984639
H	5.619560	0.220404	1.554860
H	0.407868	3.458551	-1.953456
H	1.495070	2.061691	-1.902162
H	1.134649	2.964788	-0.409405

(S,S)-MeSO C-1 Mode 2

TS

C	-0.770152	-0.636610	0.027251
O	1.246536	-0.729387	-0.465836
H	-0.873542	-1.421915	-0.712902
C	-0.941958	-1.096637	1.415136
H	-0.735174	-0.331948	2.172712
C	-0.277762	-2.406209	1.786608
C	-1.023696	0.733461	-0.437508
C	-0.932554	1.843119	0.413550
H	-0.677046	1.711078	1.460056
C	-1.173380	3.121933	-0.077646
H	-1.104242	3.976564	0.588851
C	-1.500761	3.307167	-1.422368
H	-1.686819	4.307672	-1.802287
C	-1.588335	2.208493	-2.276950
H	-1.843216	2.348572	-3.323236
C	-1.350291	0.928250	-1.785535
H	-1.420016	0.068894	-2.447542
O	-2.316434	-1.187556	1.115575
O	1.932899	0.401352	1.332729
C	2.151364	-0.161710	0.230227
C	3.578556	-0.170217	-0.299402
H	4.192506	-0.657839	0.468040
H	3.908861	0.875368	-0.327678
C	3.788686	-0.833341	-1.653445
H	3.493959	-1.887569	-1.633707
H	3.212483	-0.331806	-2.437651
H	4.847138	-0.785420	-1.931126
O	4.068024	1.570797	2.617827
H	3.309206	1.147988	2.149731
H	4.829463	1.379819	2.052817
O	0.821747	-1.981033	-2.912226
H	1.081146	-1.522029	-2.085091
H	-0.140799	-1.892388	-2.939978
O	-3.772153	0.924825	1.912845
H	-3.370400	1.704309	1.504145
H	-3.202972	0.166083	1.609902
O	-3.018547	-3.411437	-0.253570
H	-2.185662	-3.766951	-0.592471
H	-2.747058	-2.608856	0.266667
H	-0.732264	-2.795835	2.704356
H	0.792680	-2.260873	1.966797
H	-0.403124	-3.149591	0.991333

PS

C	-0.305890	0.602490	0.029195
O	1.133830	0.611555	0.352765
H	-0.642792	1.460024	0.614125
C	-0.639601	0.953136	-1.436736
H	-0.471467	0.073857	-2.077767
C	0.273453	2.076024	-1.937286
C	-0.952556	-0.634581	0.617031
C	-1.237262	-1.781240	-0.129954
H	-1.000055	-1.816221	-1.188397
C	-1.833680	-2.888323	0.471303
H	-2.055455	-3.768877	-0.125308
C	-2.143919	-2.868239	1.831239
H	-2.608483	-3.732408	2.297823
C	-1.858827	-1.731559	2.586841
H	-2.100591	-1.703624	3.645637
C	-1.271607	-0.623267	1.979049
H	-1.059746	0.267349	2.567404
O	-1.988291	1.323049	-1.468251
O	1.639727	-1.050569	-1.067348
C	1.978229	-0.235534	-0.216024
C	3.400291	-0.121208	0.268127
H	4.014847	-0.005729	-0.632117
H	3.653596	-1.105607	0.680499
C	3.701263	0.979596	1.275203
H	3.472940	1.969354	0.867490
H	3.134712	0.842974	2.201384
H	4.766090	0.957593	1.527092
O	3.664212	-2.809497	-1.947190
H	2.947642	-2.205346	-1.663883
H	4.415878	-2.556443	-1.393319
O	1.025611	2.943831	2.244966
H	1.246946	2.173753	1.695519
H	0.062117	3.010469	2.184106
O	-3.585763	-0.602079	-2.221388
H	-3.419938	-1.345726	-1.625905
H	-2.948820	0.121824	-1.908662
O	-2.365453	3.527393	-0.054987
H	-1.531791	3.664204	0.416082
H	-2.210013	2.689242	-0.596739
H	-0.069762	2.396803	-2.927116
H	1.318382	1.757975	-2.031434
H	0.235414	2.941318	-1.262976

(S,S)-MeSO C-2 Mode 1

TS

O	1.290779	-1.791638	-0.030318
C	-0.752793	-1.882280	0.329084
H	-0.616001	-1.487443	1.327954
C	-0.866777	-3.360878	0.163098
C	-1.269062	-0.950496	-0.677583
H	-1.248327	-1.364626	-1.693739
O	-2.503095	-1.174495	-0.027750
C	-0.793219	0.481650	-0.698195
C	-0.550958	1.104678	-1.923839
H	-0.665242	0.536534	-2.844826
C	-0.172721	2.446583	-1.981303
H	0.013558	2.915381	-2.943605
C	-0.041087	3.182687	-0.805873
H	0.249938	4.228675	-0.845314
C	-0.288006	2.569763	0.424267
H	-0.190724	3.138731	1.345087
C	-0.660281	1.229405	0.477366
H	-0.855624	0.769293	1.441982
C	3.281484	-0.484985	0.103479
O	1.703655	-0.746712	1.898954
H	3.979578	-0.227908	0.905306
H	3.742841	-1.253102	-0.525453
C	2.972823	0.756621	-0.742266
H	2.309837	0.510240	-1.577329
H	2.491539	1.535390	-0.140855
H	3.899340	1.169818	-1.154000
O	1.477678	-1.941961	-2.769897
H	1.449617	-1.865011	-1.788574
H	0.972041	-1.177997	-3.080129
O	2.414321	1.785244	2.770006
H	2.201032	0.880057	2.447151
H	2.069574	2.369221	2.080071
O	-4.040989	1.083931	-0.509444
H	-3.369250	1.772826	-0.616084
H	-3.516198	0.270830	-0.319600
O	-3.055781	-0.423117	2.548590
H	-2.936853	0.535232	2.595770
H	-2.796411	-0.654613	1.622891
C	2.011529	-1.046357	0.717907
H	-0.790839	-3.650405	-0.888806
H	-1.828984	-3.701405	0.556404
H	-0.082578	-3.858087	0.737729

PS

O	1.022850	-1.574984	-0.092878
C	-0.353209	-1.813071	0.336736
H	-0.441802	-1.488145	1.374508
C	-0.606433	-3.300148	0.216707
C	-1.265315	-0.954914	-0.564348
H	-1.140257	-1.345717	-1.592078
O	-2.594076	-1.072358	-0.151713
C	-0.801371	0.500282	-0.593112
C	-0.558798	1.136326	-1.812420
H	-0.659071	0.571977	-2.737621
C	-0.194929	2.482959	-1.863120
H	-0.009723	2.957922	-2.822953
C	-0.069610	3.215367	-0.684321
H	0.213835	4.263751	-0.717815
C	-0.313651	2.592428	0.541503
H	-0.222975	3.156975	1.466100
C	-0.676331	1.248080	0.584546
H	-0.867249	0.779180	1.546742
C	3.172883	-0.615917	0.035853
O	1.654613	-0.766080	1.906689
H	3.931726	-0.452640	0.804272
H	3.492034	-1.443506	-0.604967
C	2.976792	0.652656	-0.806393
H	2.268343	0.479687	-1.621111
H	2.604635	1.481120	-0.194205
H	3.934915	0.951868	-1.241480
O	1.383246	-1.898355	-2.949928
H	1.302812	-1.799721	-1.984302
H	0.849092	-1.172078	-3.301121
O	2.492274	1.849509	2.700916
H	2.268720	0.925901	2.476410
H	2.086006	2.363837	1.988645
O	-3.975408	1.164695	-0.513634
H	-3.311939	1.866672	-0.570182
H	-3.433820	0.324679	-0.391459
O	-3.042467	-0.874613	2.438095
H	-2.772111	0.019160	2.688101
H	-2.822348	-0.937928	1.454948
C	1.894130	-0.994932	0.726718
H	-0.484008	-3.631982	-0.820842
H	-1.626787	-3.528020	0.537480
H	0.086775	-3.860202	0.852471

(S,S)-MeSO C-2 Mode 2

TS

C	-2.100867	-0.692188	2.667934
H	-3.026710	-0.780337	3.229458
C	-1.257801	-1.792865	2.526908
H	-1.519562	-2.743967	2.982349
C	-1.746806	0.526543	2.086857
H	-2.399038	1.389387	2.194175
C	-0.073161	-1.674126	1.799371
H	0.583796	-2.535013	1.694202
C	-0.564385	0.639668	1.360149
H	-0.307541	1.591592	0.905020
C	0.280556	-0.463061	1.200708
C	1.546816	-0.383215	0.384856
H	2.030804	-1.369208	0.362436
O	2.450754	0.646571	0.712334
C	1.506843	0.247240	-0.943909
H	0.970287	1.186316	-1.011218
C	2.537415	-0.050444	-1.980478
O	-0.062832	-0.722622	-1.840060
C	-1.178582	-0.094770	-1.723373
O	-1.288573	1.133103	-1.941192
C	-2.371801	-0.930818	-1.296511
H	-2.505644	-1.714502	-2.052908
H	-2.078692	-1.450629	-0.375553
C	-3.663940	-0.154510	-1.086454
H	-4.462822	-0.834808	-0.772476
H	-3.986589	0.345332	-2.005793
H	-3.546559	0.607760	-0.308912
H	4.024921	0.021455	0.594354
O	4.939308	-0.364946	0.531604
H	4.992052	-0.706834	-0.371417
H	1.964249	2.320324	0.819355
O	1.794974	3.294776	0.861857
H	1.006306	3.426005	0.317625
H	0.078939	-2.429218	-1.447997
O	0.200356	-3.392847	-1.278258
H	-0.085610	-3.510817	-0.362098
H	-1.961233	2.453331	-0.915581
O	-2.218920	3.275776	-0.447298
H	-2.180264	3.043048	0.490177
H	3.416672	0.579493	-1.815426
H	2.144034	0.188419	-2.970603
H	2.841932	-1.100349	-1.947400

PS

C	-2.297628	1.949095	1.693396
H	-3.258544	2.368310	1.979570
C	-1.900578	0.703817	2.179511
H	-2.550383	0.146536	2.849123
C	-1.449332	2.655802	0.838257
H	-1.751151	3.627666	0.456067
C	-0.664386	0.172979	1.810082
H	-0.359434	-0.798853	2.193031
C	-0.220601	2.114646	0.463865
H	0.429028	2.664162	-0.212478
C	0.184966	0.864814	0.941349
C	1.494358	0.238817	0.468247
H	1.772803	-0.531309	1.213969
O	2.518605	1.162231	0.305758
C	1.247268	-0.533674	-0.856319
H	0.884007	0.153560	-1.623835
C	2.458191	-1.298444	-1.345723
O	0.207481	-1.526211	-0.605717
C	-1.057542	-1.282970	-0.949623
O	-1.389481	-0.326389	-1.635455
C	-1.994171	-2.335059	-0.423205
H	-1.804362	-3.246356	-1.005070
H	-1.699268	-2.560723	0.606645
C	-3.459976	-1.933223	-0.499965
H	-4.083518	-2.743253	-0.109752
H	-3.768287	-1.732221	-1.530308
H	-3.652300	-1.036466	0.098619
H	3.906621	0.439860	0.633537
O	4.783465	-0.001141	0.885712
H	4.832566	-0.786485	0.324501
H	2.539531	1.919993	-1.128086
O	2.593071	2.418040	-2.005054
H	1.719062	2.295667	-2.399981
H	0.700649	-2.701223	0.853579
O	1.004284	-3.270690	1.583097
H	1.448278	-2.657274	2.185330
H	-2.924675	0.746661	-1.597151
O	-3.622228	1.430229	-1.594336
H	-3.476783	1.903255	-0.762122
H	3.260697	-0.599969	-1.596556
H	2.208851	-1.871309	-2.244662
H	2.821608	-1.988386	-0.575233

S7. References

1 Bauer, P.; Janfalk Carlsson, Å.; Amrein, B.; Dobritzsch, D.; Widersten, M.; Kamerlin, S. C. L. *Org. Biomol. Chem.* **2016**, *14*, 5639-5651.

2 Amrein, B. A.; Bauer, P.; Duarte, F.; Janfalk Carlsson, Å.; Naworyta, A.; Mowbray, S. L.; Widersten, M.; Kamerlin, S. C. L. *ACS Catal.* **2015**, *5*, 5702-5713.

3 Warshel, A.; Weiss, R. M. *J. Am. Chem. Soc.* **1980**, *102*, 6218-6226.

4 Jorgensen, W. L.; Maxwell, D. S.; Tirado-Rives, J. *J. Am. Chem. Soc.* **1996**, *118*, 11225-11236.

5 Schrödinger Release 2013-3: MacroModel version 9.1, 2013, Schrödinger LLC, New York.

6 Cieplak, P.; Cornel, W. D.; Bayly, C.; Kollman, P. A. *J. Comput. Chem.* **1995**, *16*, 1357-1377.

7 Frisch, G. W.; Schlegel, H. B.; Scuseria, G. E.; Robb, M. A.; Cheeseman, J. R., *et al., Gaussian 09 Rev. C01*, 2009, Gaussian Inc., Wallingford CT.

8 Wang, J.; Wang, W.; Kollman, P. A.; Case, D. A. *J. Mol. Graph. Mod.* **2006**, *25*, 247-260.

9 Marelius, J.; Kolmodin, K.; Feierberg. I.; Åqvist, J. *J. Mol. Graph. Mod.* **1998**, *16*, 213-225.

10 Abraham, M. J.; Murtola, T.; Schulz, R.; Páll, S.; Smith, J. C.; Hess, B.; Lindahl, E. *SoftwareX* **2015**, *1-2*, 19-25.

11 Berman, H. M.; Westbrook, J.; Feng, Z.; Gilliland, G.; Bhat, T. N.; Weissig, H.; Shindyalov, I. N.; Bourne, P. E. *Nucleic Acids Res.* **2000**, *28*, 235-242.

12 Mowbray, S. L.; Elfström, L. T.; Ahlgren, K. M.; Andersson, C. E.; Widersten, M. *Protein Sci.* **2006**, *15*, 1628-1637.

13 Janfalk Carlsson, Å.; Bauer, P.; Dobritzsch, D.; Nilsson, M.; Kamerlin, S. C. L.; Widersten, M. *ChemBioChem* **2016**, *17*, 1693-1697.

14 Jorgensen, W. L.; Chandrasekhar, J.; Madura, J. D.; Impey, R. W.; Klein, M. L. *J. Chem. Phys.* **1983**, *79*, 926-935.

15 Ryckaert, J.-P.; Ciccotti, G.; Berendsen,, H. J. C. *J. Comput. Phys.* **1977**, *23*, 327-341.

16 Olsson, M. H. M.; Sondergaard, C. R.; Rostkowski, M.; Jensen, J. H. *J. Chem. Theory Comput.* **2011**, *7*, 525-537.

17 Sondergaard, C. R.; Olsson, M. H. M.; Rostkowski, M.; Jenssen, J. H. *J. Chem. Theory Comput.* **2011**, *7*, 2284-2295.

18 Barrozo, A.; Duarte, F.; Bauer, P.; Carvalho; A. T. P.; Kamerlin, S. C. L. *J. Am. Chem. Soc.* **2015**, *137*, 9061-9076.

19 Blaha-Nelson, D.; Krüger, D. M.; Szeler, K.; Ben-David, M.; Kamerlin, S. C. L. *J. Am. Chem. Soc.* **2017**, *139*, 1155-1167.

20 Chen, V. B.; Arendall, III. W. B.; Headd, J. J.; Keedy, D. A.; Immormino, R. M.; Kapral, G. J.; Murray, L. W.; Richardson, J. S.; Richardson, D. C. *Acta Cryst.* **2010**, *D66*, 12-21.

21 Berendsen, H. J. C.; Postma, J. P. M.; van Gunsteren, W. F.; DiNola, A.; Haak, J. R. *J. Chem. Phys.* **1984**, *81*, 3684-3690.

22 Lee, F. S.; Warshel, A. *J. Chem. Phys.* **1992**, *97*, 3100-3107.

23 Warshel, A. **1991** *Computer Modeling of Chemical Reactions in Enzymes and Solutions*, Wiley, New York.

24 Shurki, A.; Derat, E.; Barrozo, A.; Kamerlin, S. C. L. *Chem. Soc. Rev.* **2015**, *44*, 1037-1052.

25 Hratchian, H. P.; Schlegel, H. B. *J. Chem. Phys.* **2004**, *120*, 9918-9924.

26 Hratchian, H. P.; Schlegel, H. B. *J. Chem. Theory Comput.* **2005**, *1*, 61-69.

27 Hong, G.; Rosta, E.; Warshel, A. *J. Phys. Chem. B* **2006**, *110*, 19570-19574.
